# Gene therapy using genome‐edited iPS cells for targeting malignant glioma

**DOI:** 10.1002/btm2.10406

**Published:** 2022-09-10

**Authors:** Ryota Tamura, Hiroyuki Miyoshi, Kent Imaizumi, Masahiro Yo, Yoshitaka Kase, Tsukika Sato, Mizuto Sato, Yukina Morimoto, Oltea Sampetrean, Jun Kohyama, Munehisa Shinozaki, Atsushi Miyawaki, Kazunari Yoshida, Hideyuki Saya, Hideyuki Okano, Masahiro Toda

**Affiliations:** ^1^ Department of Neurosurgery Keio University School of Medicine Shinjuku‐ku, Tokyo Japan; ^2^ Department of Physiology Keio University School of Medicine Shinjuku‐ku, Tokyo Japan; ^3^ Laboratory for Cell Function and Dynamics, RIKEN Center for Brain Science Wako, Saitama Japan; ^4^ Department of Geriatric Medicine Graduate School of Medicine, The University of Tokyo Bunkyo‐ku, Tokyo Japan; ^5^ Division of Gene Regulation Keio University School of Medicine Shinjuku‐ku, Tokyo Japan

**Keywords:** CRISPR/Cas9, ferroptosis, gene therapy, glioblastoma, migration

## Abstract

Glioblastoma is characterized by diffuse infiltration into the normal brain. Invasive glioma stem cells (GSCs) are an underlying cause of treatment failure. Despite the use of multimodal therapies, the prognosis remains dismal. New therapeutic approach targeting invasive GSCs is required. Here, we show that neural stem cells (NSCs) derived from CRISRP/Cas9‐edited human‐induced pluripotent stem cell (hiPSC) expressing a suicide gene had higher tumor‐trophic migratory capacity compared with mesenchymal stem cells (MSCs), leading to marked in vivo antitumor effects. High migratory capacity in iPSC‐NSCs was related to self‐repulsive action and pathotropism involved in EphB‐ephrinB and CXCL12‐CXCR4 signaling. The gene insertion to *ACTB* provided higher and stable transgene expression than other common insertion sites, such as *GAPDH* or *AAVS1*. Ferroptosis was associated with enhanced antitumor immune responses. The thymidylate synthase and dihydroprimidine dehydrogenase expressions predicted the treatment efficacy of therapeutic hiPSC‐NSCs. Our results indicate the potential benefit of genome‐edited iPS cells based gene therapy for invasive GSCs. Furthermore, the present research concept may become a platform to promote clinical studies using hiPSC.

## 1 INTRODUCTION

Glioblastoma is characterized by diffuse infiltration into the normal brain parenchyma.^1^, ^2^ Remarkable invasiveness is responsible for its recurrence after surgical resection. In addition to differentiated glioma cells, the presence of glioma stem cells (GSCs), which have self‐renewal and tumor‐initiating capacity, is also an underlying cause of invasiveness and resistance to chemoradiotherapy.^3^, ^4^, ^5^ Neural stem cells (NSCs) and mesenchymal stem cells (MSCs) have tumor‐trophic migratory capacity and were previously used as therapeutic gene delivery vehicles (GDVs) for glioblastomas.^6^, ^7^ NSCs from human embryos have ethical and practical difficulties.^8^ Human‐induced pluripotent stem cells (hiPSCs) differentiate into NSCs, circumventing ethical and practical issues.^9^, ^10^, ^11^, ^12^ Whether NSCs or MSCs are useful GDVs for diffuse infiltrative glioma cell treatment is unclear.

Suicide gene therapy using herpes simplex virus thymidine kinase (HSVtk) or cytosine deaminase (CD) might be appropriate for glioblastoma.^13^ However, transduced HSVtk was silenced or cytotoxic during neuronal differentiation of hiPSCs.^14^ Stable constitutive expression of therapeutic genes in hiPSCs is difficult using viral vectors,^15^, ^16^ which integrate randomly into host genomes, which raises concerns of insertional mutagenesis and oncogene activation.^17^ Therefore, stable and safe transgene expression is required by designed insertion into the appropriate loci using genome‐editing technology.^18^

We established a novel therapeutic approach using genome‐edited iPSC‐derived NSCs targeting invasive glioblastomas.

## 2 RESULTS

### 2.1 Tumor‐trophic migratory capacity of NSCs

Migratory capacity was evaluated using time‐lapse imaging of organotypic brain slice cultures (Figure S1A). hiPSC‐NSCs and fetal NSCs (FNSCs) expressing Kusabira‐Orange with humanized codon (hKO1) exhibited directional migration toward U87 glioma‐derived tumors expressing ffLuc (fusion protein consisting of Aequorea GFP [Venus] and firefly luciferase^19^) compared with human adipose‐derived MSCs (AMSCs) and bone marrow‐derived MSCs (BMSCs; Figure S1B). Rose diagram map shows the spatial distribution of NSCs and MSCs around the tumor, including both the number of cells in various directions and their distance toward the tumor center. hiPSC‐NSCs (hKO1^+^) demonstrated preferential localization around the tumor compared with MSCs (Figure S1C). Y‐axis positive components of hiPSC‐NSCs (hKO1^+^) were significantly longer than negative components (Figure S1D). No differences in migration speeds between hiPSC‐NSCs (hKO1^+^) and other stem cells were observed. Net distance of hiPSC‐NSCs (hKO1^+^) was significantly longer than other stem cells. Pseudopods of hiPSC‐NSCs (hKO1^+^) were significantly longer than other stem cells except BMSC1 and 2 (Figure S1E). hiPSC‐NSCs (hKO1^+^) migrated toward tumors on the contralateral side through the corpus callosum (Figure S2A,B). hiPSC‐NSCs (hKO1^+^) migrated along diffusely infiltrative hG008 (ffLuc) GSCs (Figure S2C; Movie S1). hiPSC‐NSC (hKO1^+^) migrated faster in slice cultures from brains transplanted with hiPSC‐NSCs (hKO1^+^) and hG008 (ffLuc) than brains transplanted with U87 cells (ffLuc; Figure S2D).

hiPSC‐NSC migration toward glioma cells was determined by optical clearing of brain tissues after hG008 (ffLuc) and hiPSC‐NSCs (hKO1^+^) implantation (Figure S2E). Three‐dimensional images of striatum revealed that iPS‐NSCs (hKO1^+^) had long pseudopods along the z‐axis, similar to hG008 cells (Figure S2F).^4^ In hG008 (ffLuc) cell‐implanted brains, hiPSC‐NSCs (hKO1^+^) trafficked to the hG008 cell (ffLuc) invasion (Figure S2G).

### 2.2 Tumor‐supportive effect of MSCs

U87 cell (ffLuc) numbers increased when cultured with MSCs compared with hiPSC‐NSCs or FNSCs (Figure S3A). U87 cells (ffLuc) had multipolar or monopolar pseudopods in the presence or absence of MSCs, respectively (Figure S3B; Movie S2). C‐X‐C motif chemokine ligand (CXCL) 1, CXCL 12, macrophage migration inhibitory factor (MIF), interleukin (IL)‐6 and IL‐8 expressions increased in MSCs co‐cultured with U87 cells (ffLuc; Figure S3C). α‐Smooth muscle actin (αSMA) and fibroblast activation protein (FAP), markers of cancer‐associated fibroblasts (CAFs), were detected in all MSCs. U87 mixed‐culture conditions significantly increased FAP^+^ cell percentages in all MSCs (Figure S3D). After MSC implantation, FAP^+^/hKO1^−^ fibroblasts concentrated around MSCs (Figure S3D).

### 2.3 Tumor‐trophic migration signaling

**FIGURE 1:**
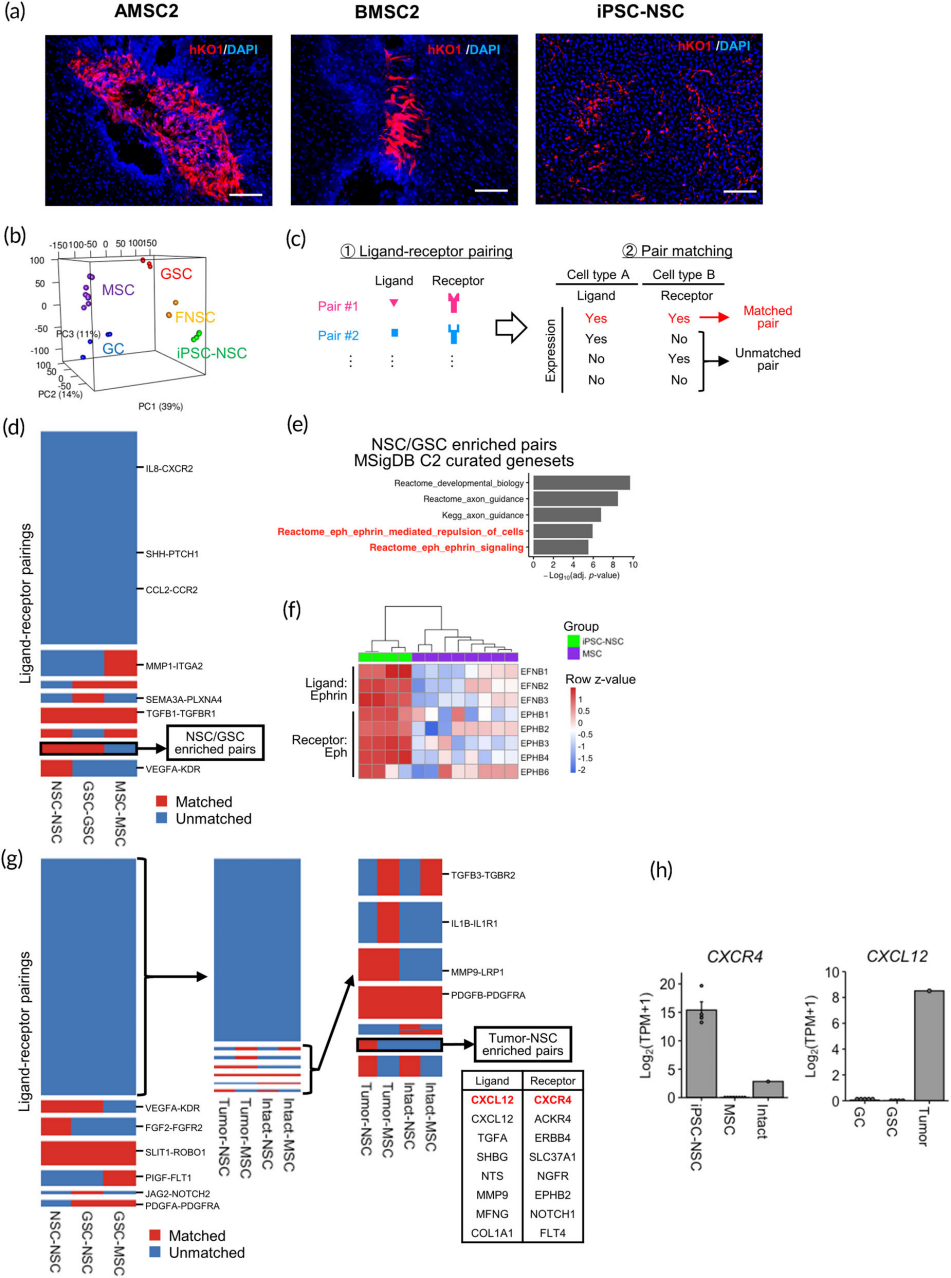
RNA‐seq data analysis. (a) Analysis of mice implanted with iPSC‐NSC, AMSC2, or BMSC2 (hKO1^+^) in the absence of U87 cells (ffLuc). Scale bar, 100 μm. (b) 3D plot of principal component analysis. Each dot represents a sample. (c) Overview of the in silico ligand–receptor pairing analysis. All curated ligand–receptor pairs were screened and pair matching was extracted based on the presence of a complementary ligand or receptor. (d) Heatmap showing the matched or unmatched status of autocrine cell–cell interactions for each cell type for all curated ligand–receptor pairs. (e) Enrichment analysis of NSC/GSC‐specific autocrine ligand‐receptor pairs across C2 genesets in MSigDB. (f) Heatmap of EphB/ephrinB gene expression. (g) Heatmap showing the matched or unmatched status of paracrine cell–cell interactions for each cell type for ligand‐receptor pairs. (Left) Status of paracrine cell–cell interactions between NSCs/MSCs and GSCs for all curated pairs. (Middle and Right) Status of paracrine cell–cell interactions between NSCs/MSCs and tumor/intact brain tissue for selected pairs. A list of eight ligand–receptor pairs specific to the tumor–NSC interaction is shown. (h) The expression level of *CXCR4* and *CXCL12*

In the absence of U87 cells, MSCs (hKO1^+^) formed clumps whereas hiPSC‐NSCs (hKO1^+^) exhibited diffuse engraftment (Figure 1a). To reveal genes related to tumor‐trophic migration, RNA‐seq analysis was performed in iPSC‐NSCs, FNSCs, AMSCs, BMSCs, glioma cell lines (GC; U87, U251, SF126) and GSCs (hG008, hG020). Principal component analysis revealed that the iPSC‐NSC gene expression profile was similar to FNSCs but distinct from MSCs (Figure 1b). GCs and GSCs have different invasive characteristics.^4^ Gene ontology analysis of NSCs and MSCs revealed that upregulated genes in NSCs were primarily enriched with terms related to “neuronal axon” and “synapse,” whereas downregulated genes were associated with “extracellular organization” (Figure S4A).

In silico ligand–receptor pairing analysis based on RNA‐seq datasets was used to identify signals underlying NSC‐specific tumor tropism. We screened curated ligand–receptor pairs and determined potential interactions for each cell type related to the presence of a complementary ligand or receptor in every other cell type (Figure 1c). We evaluated autocrine interactions to detect self‐repulsion signaling pathways because cell behavior differed between NSCs and MSCs, even without tumor cells (Figure 1a). GSCs displayed a diffusive invasion pattern, similar to the migration of NSCs. Ninety‐two ligand–receptor pairs were autocrine active in iPSC‐NSCs and GSCs, but not MSCs, and were associated with Eph/ephrin signaling (Figure 1d,e). Eph/ephrin activation generates self‐repulsive signals and EphB/ephrinB signaling is linked to glioblastoma invasion.^20^ Most EphB/ephrinB genes were upregulated in NSCs compared with MSCs (Figure 1f), which suggests that EphB/ephrinB signaling enables diffuse NSC migration compared with MSCs.

The paracrine interactions between GSCs and NSCs/MSCs were evaluated to identify the potential signaling for chemoattraction toward GSCs. Analysis of 27 ligand–receptor pairs activated in GSCs and NSCs (Figure 1g) demonstrated no enrichment of specific signaling pathways. This result led us to hypothesize that the gene expression profile of in vitro‐cultured GSCs did not mirror the precise physiology of intracerebral glioblastoma, hindering the identification of chemoattractive signaling from glioblastoma. The addition of glioblastoma and parental intact brain tissue RNA‐seq data to our in silico ligand–receptor pairing analysis has indicated that eight ligand–receptor pairs were selectively active between resected glioblastoma and NSCs (Figure 1g). The analysis suggested that NSCs, but not MSCs, migrated toward glioblastomas by EphB‐ephrinB and C‐X‐C motif chemokine ligand (CXCL)12‐ C‐X‐C motif chemokine receptor (CXCR)4 signaling pathways. We focused on CXCL12‐CXCR4 because it regulates tumor tropism.^21^ The interactions involving chemoattractant chemokine ligands and receptors are activated in the microenvironment of brain tumors.^22^ *CXCR4* was highly expressed in NSCs but not in MSCs; *CXCL12* ligand expression was upregulated in resected glioblastoma, compared with in vitro‐cultured tumor‐derived cells (Figure 1h). Reanalysis of published RNA‐seq data of resected glioblastoma demonstrated that *CXCL12* was expressed in most samples^23^, ^24^, ^25^ (Figure S4C). Furthermore, we have performed the migration assay using a CXCR4 antagonist (AMD3100) and a specific EphB4 inhibitor (NVP‐BHG712). Although no significant change of net distance was observed in NSC (*p* = 0.32), antagonist for CXCR4 have blocked CXCL12‐mediated iPSC‐NSC pathotropism toward glioma cells (Figure S4D). On the other hand, the inhibition of EphB4 had little effect on pathotropism, but net distance was significantly decreased (*p* = 0.026). Inhibition of EphB/ephrinB pathway has blocked self‐repulsive action (Figure S4E). This result has suggested the importance of the CXCL12/CXCR4 and EphB/ephrinB pathway in regulating homing of engrafted NSCs to malignant glioma sites.

### 2.4 Lentiviral vector‐mediated transduction

We evaluated efficiency of lentiviral vector‐mediated transduction in hiPSCs and NSCs. hiPSCs were transduced with the lentiviral vector CSII‐EF‐yCD‐uracil phosphoribosyltransferase (UPRT)‐IRES‐hKO1 at a MOI of 2 (Figure S5A). hiPSCs with yCD‐UPRT‐hKO1 was subsequently differentiated into NSCs (Figure S5B). hiPSCs were sensitive to 5‐fluorocytosine (5‐FC). However, transduced yCD‐UPRT‐hKO1 was silenced during neuronal differentiation of hiPSCs. hKO1 fluorescence signal was not detected in NSCs (Figure S5B). NSCs were all 5‐FC resistant (Figure S5B). NSCs derived from hiPSC were transduced with the lentiviral vector CSII‐EF‐yCD‐UPRT‐IRES‐hKO1 at a MOI of 2 (CD‐NSC [Lenti]). Although 70%–80% of NSCs were hKO1‐positive immediately after transduction, the proportion of hKO1‐positive cells decreased with time, and <5% of NSCs were hKO1‐positive after the second passage (Figure S5C). No yCD‐UPRT expression‐induced cytotoxicity was observed in hiPSC compared with HSVtk^12^, ^14^ but transgene silencing occurred during NSC induction and passage even when hiPSC‐derived NSCs were transduced with lentiviral vector.

### 2.5 Gene loci for yCD‐UPRT expression

**FIGURE 2:**
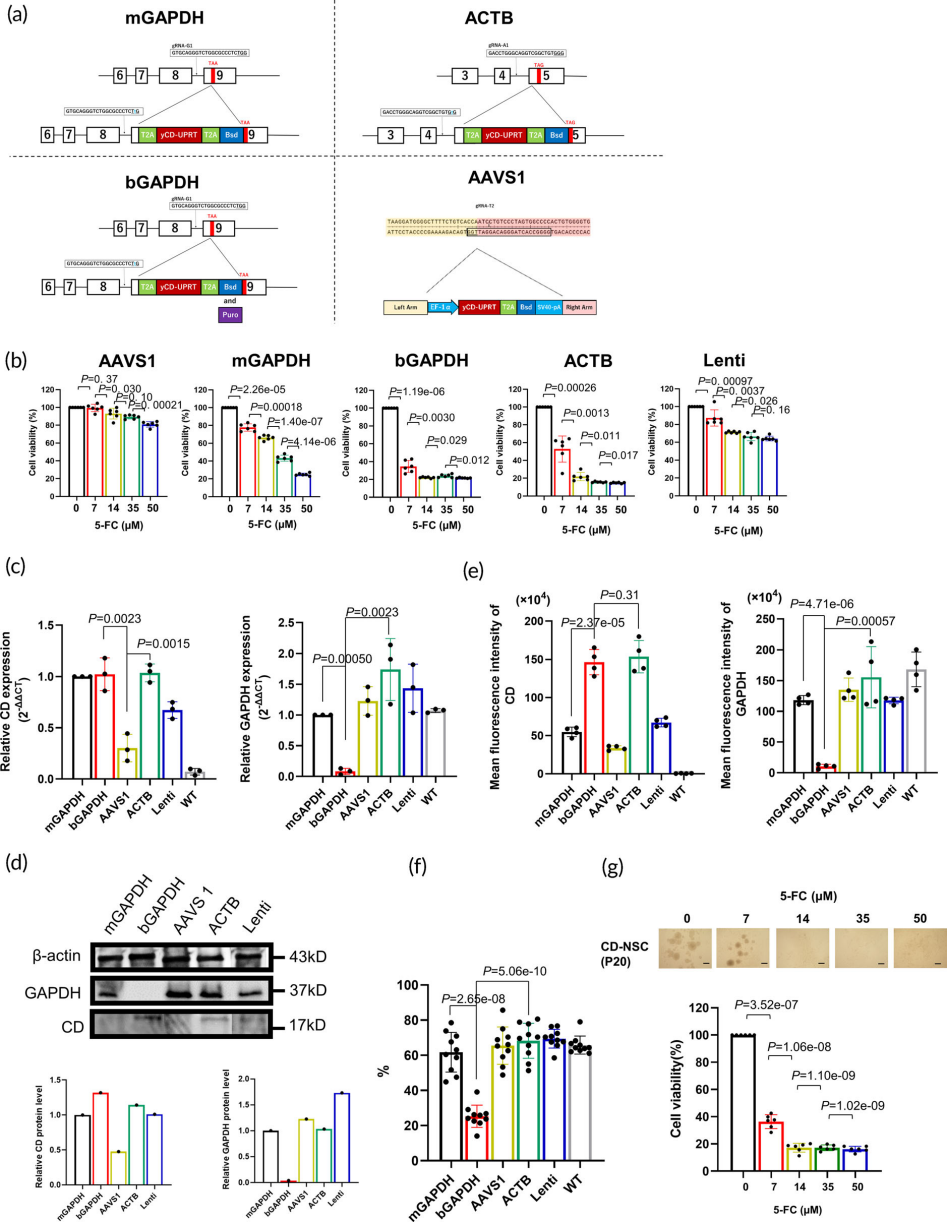
Gene loci achieving high and stable yCD‐UPRT expression. (a) Schematic depiction of the CRISPR/Cas9‐mediated strategy for inserting the *yCD‐UPRT* gene into the indicated locus. Single guide RNA (sgRNA) target sequence and HR donor constructs are shown. (b) Each CD‐NSC was cultured in the presence of 0, 7, 14, 35, or 50 μM 5‐FC for 7 days. The sensitivity to 5‐FC was evaluated by CCK‐8 assay. Data represent the mean ± SD (*n* = 6). (c) qRT‐PCR analysis of each CD‐NSC for *CD* or *GAPDH* expression. Gene expression was normalized relative to β‐actin expression. Data represent the mean ± SD (*n* = 3). (d) Western blot analysis of each CD‐NSC for CD or GAPDH expression. β‐Actin was used as an internal control. (e) Immunocytochemical analysis of neurospheres stained with anti‐GAPDH antibody or anti‐CD antibody and DAPI. Densitometry of signals was evaluated. (f) Immunocytochemical analysis of EdU^+^ cells (red) in neurospheres. Nuclei were stained with Hoechst 33258 (blue). The frequency of neurospheres containing immunopositive cells is shown. Data represent the mean ± SD (*n* = 10). (g) Representative images of CD‐NSC (ACTB; at passage 20). CD‐NSCs (ACTB) were cultured in the presence of 0, 7, 14, 35 or 50 μM 5‐FC for 7 days. The sensitivity to 5‐FC was evaluated by CCK‐8 assay. Data represent the mean ± SD (*n* = 3). Scale bar, 100 μm. WT, wild‐type iPSC‐derived NSCs

To overcome *yCD‐UPRT* silencing in hiPSCs by lentiviral vector, we used CRISPR/Cas9‐mediated genome editing. *yCD‐UPRT* was inserted into monoallelic *GAPDH* (mGAPDH), biallelic *GAPDH* (bGAPDH), monoallelic *ACTB*, or *AAVS1* loci (Figure 2a; Table S1). CD‐NSC (bGAPDH and ACTB) showed higher sensitivity to 5‐FC than other CD‐NSCs (Figure 2b; Figure S6A). Although iPSCs with yCD‐UPRT transduced into the AAVS‐1 locus were sensitive to 5‐FC, CD‐NSCs (AAVS) were 5‐FC resistant, which suggests transgene silencing. yCD expression in CD‐NSCs (bGAPDH and ACTB) was significantly higher than in other CD‐NSCs (Figure 2c). Significantly lower *GAPDH* expression in CD‐NSCs (bGAPDH) than in other CD‐NSCs was confirmed by quantitative reverse transcription PCR, western blotting, and immunocytochemistry (Figure 2c–e; Figure S6B). Decreased *GAPDH* expression in CD‐NSCs (bGAPDH) was associated with neurosphere growth rate. Neurospheres formed by CD‐NSCs (bGAPDH) were significantly smaller than CD‐NSCs (ACTB). It was difficult to maintain and subculture CD‐NSCs (bGAPDH) (Figure S6C). EdU incorporation into neurospheres was significantly lower in CD‐NSCs (bGAPDH) than in other CD‐NSCs (Figure 2f; Figure S6D). No differences in 5‐FC sensitivity were observed in CD‐NSCs (ACTB) between early and late passages, showing stable constitutive transgene expression (Figure 2b,g). Gene expression profiles of CD‐NSCs (ACTB) and wild‐type iPSC‐NSCs were similar (Figure S4B), which indicated that insertion to *ACTB* provided high and stable yCD‐UPRT expression.

Whole‐genome sequencing is a method to rapidly identify genetic variations. Whole‐genome sequencing showed no off‐target mutations induction in CD‐iPSCs and CD‐NSCs (Figure S6E,F).

### 2.6 Therapeutic efficacy of CD‐NSCs (ACTB) in human GSC mouse model

**FIGURE 3:**
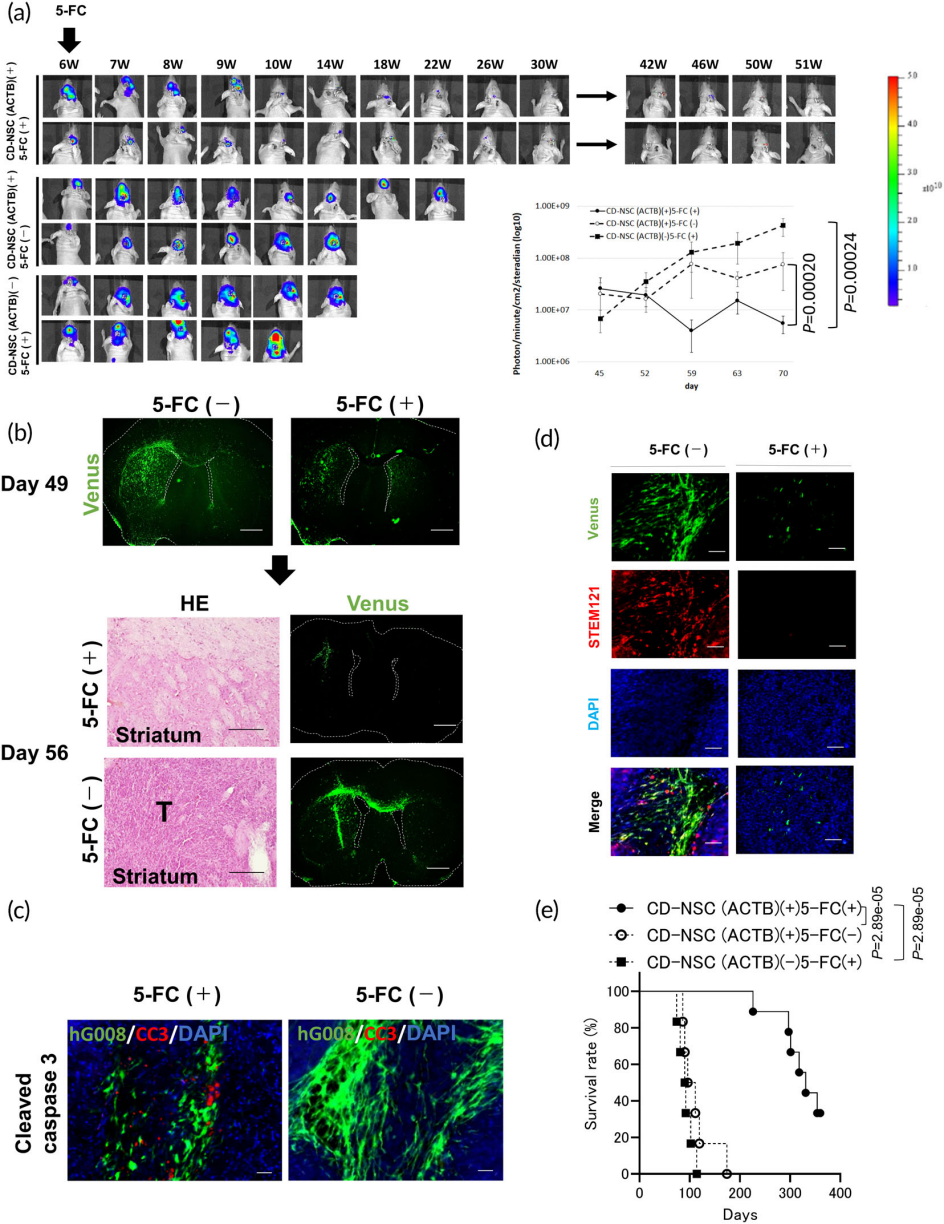
Tumor killing effect of CD‐NSC (ACTB) for human GSC model. (a) Representative BLI images and radiance intensities of mice transplanted with hG008 cells (ffLuc). The colored scale bar represents BLI radiance intensity in photons/minute/cm^2^/steradian. SD (error bars) is shown. (b–d) H&E staining and Venus fluorescence images of brain sections after 5‐FC or PBS administration at different time point (*n* = 6/group). T, tumor (b). Immunofluorescence images of cleaved caspase‐3 (c), Venus, and STEM121 (d). Scale bar, 100 μm. (e) Kaplan–Meier plots of survival of mice transplanted with hG008 cells (ffLuc; +5‐FC; *n* = 6), hG008 cells (ffLuc) and CD‐NSC (ACTB; −5‐FC; *n* = 6), and hG008 cells (ffLuc) and CD‐NSC (ACTB; +5‐FC; *n* = 9). Data are combined from two independent experiments.

Luciferase‐based bioluminescence imaging (BLI) signal intensity was decreased in mice transplanted with hG008 cells (ffLuc) and CD‐NSCs (ACTB) followed by 5‐FC (Figure 3a). Tumor cells were gradually killed by the bystander killing effect of CD‐NSCs (ACTB) (Figure 3b). H&E staining and Venus^26^‐derived fluorescence demonstrated complete tumor disappearance (2/11 mice) or reduced tumor volume in brains of treated mice compared with controls. No hG008 cells (ffLuc) were observed in contralateral brains of treated mice compared with controls (Figure 3b). Cleaved caspase‐3 immunohistochemistry demonstrated apoptotic cell death in 5‐FC‐treated mice (Figure 3c). CD‐NSCs (ACTB) expressed human‐specific cytoplasmic antigen recognized by STEM121 antibody in control mice without 5‐FC administration but not in mice with 5‐FC administration (Figure 3d). Survival of treated mice was significantly prolonged compared with controls (ffLuc; Figure 3e).

Organotypic brain slice culture was used to visualize antitumor effects on hG008 cells (ffLuc). 5‐FC reduced hG008 cell (ffLuc) growth in the slice cultures with CD‐NSCs (hKO1^+^; Figure S7A,B; Movie S3). Immunofluorescence and H&E analysis demonstrated CD‐NSCs (hKO1^+^) in the tumor core containing hG008 cells (ffLuc; Figure S7A). Temozolomide (TMZ),^1^ standard malignant glioma chemotherapy, did not suppress hG008 (ffLuc) proliferation (Figure S7A,B). Long pseudopods and nestin expression were observed in hG008 cells (ffLuc) after adding PBS and TMZ but not remnant hG008 cells (ffluc) after adding 5‐FC (Figure S7B). Antitumor effects of converted 5‐fluorouracil (5‐FU) released from CD‐NSCs were similar to those of exogenous 5‐FU on hG008, which suggests high local concentration of 5‐FU in brain parenchyma (Figure S7C).

### 2.7 Therapeutic efficacy of CD‐mNSCs in immunocompetent mouse model of GSC

**FIGURE 4:**
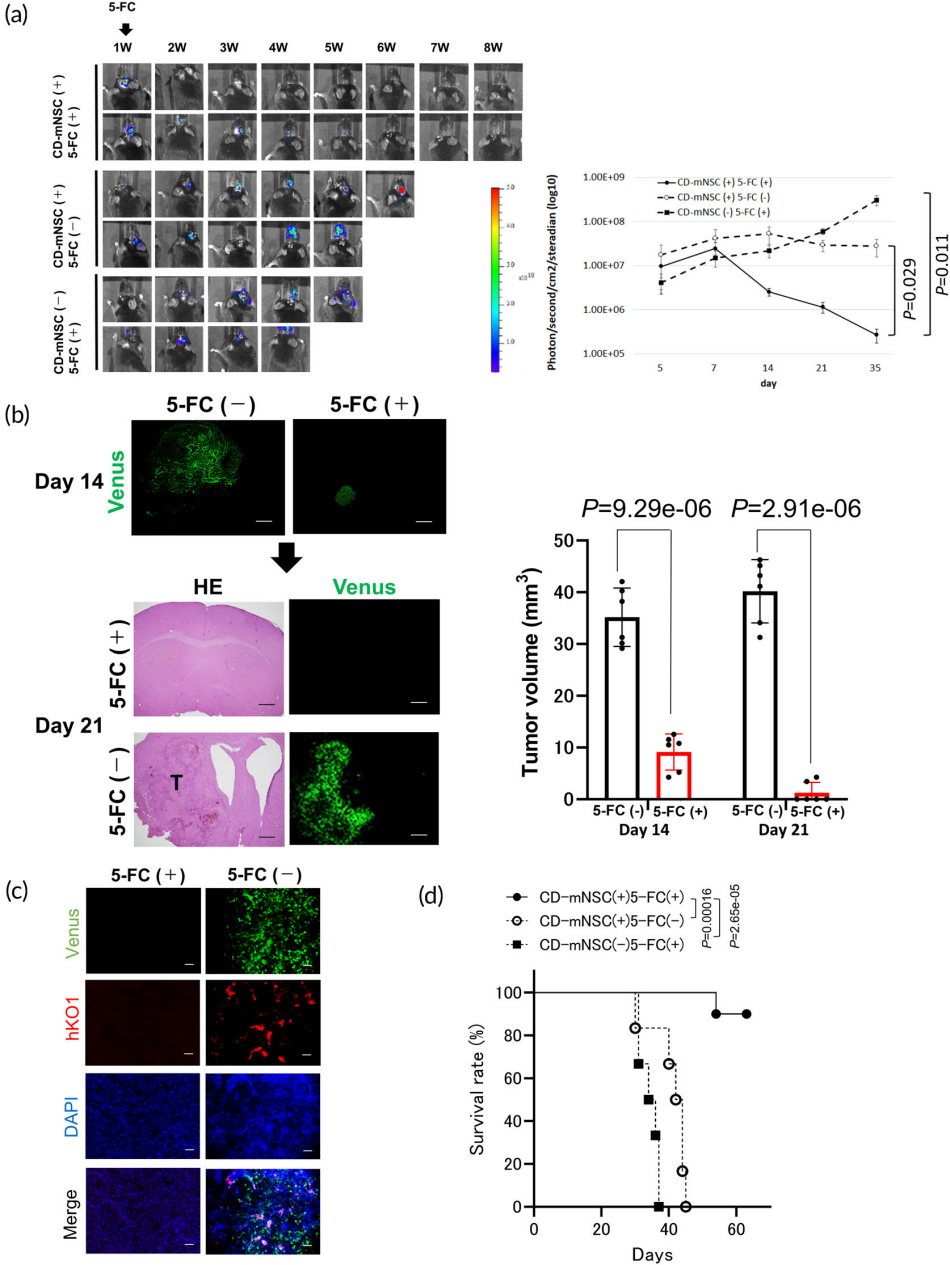
Tumor killing effect of mCD‐NSC for mouse GSC model. (a) Representative BLI images and radiance intensities of mice. The colored scale bar represents BLI radiance intensity in photons/second/cm^2^/steradian. SD (error bars) is shown. (b, c) Mice with TSG cells (ffLuc) and CD‐mNSCs were euthanized at 2 and 3 weeks after PBS or 5‐FC administration (*n* = 6/group) and tumor volume was analyzed. H&E staining and Venus fluorescence images of brain sections are shown. Data represent the mean ± SD (*n* = 6). T, tumor (b). hKO1 expression representing CD‐mNSC (c). Scale bar, 100 μm. (d) Kaplan–Meier plots showing survival of mice transplanted with TSG cells (ffLuc; +5‐FC; *n* = 6), TSG cells (ffLuc) and CD‐mNSCs (−5‐FC; *n* = 7), and TSG cells (ffLuc) and CD‐mNSCs (+5‐FC; *n* = 10). Data are combined from two independent experiments.

To evaluate T cell‐mediated antitumor immune response, NSCs differentiated from 38C2 mouse iPSC line^27^, ^28^ were transduced with the lentiviral vector CSII‐EF‐yCD‐UPRT‐IRES‐hKO1. yCD‐UPRT‐transduced mouse iPSC‐derived NSCs (CD‐mNSCs) were highly sensitive to 5‐FC. Stable constitutive *yCD‐UPRT* expression was achieved in mNSCs (Figure S8A). Representative BLI, radiance intensities, tumor volume, and Kaplan–Meier plots of treated mice indicated notable antitumor effects (Figure 4a–d; Figure S8B). Tumor cells were gradually killed by the bystander killing effect of CD‐mNSCs (Figure 4b). Histological analysis showed the complete disappearance of TSG cells (ffLuc) in 4/6 mice (Figure 4b). CD8^+^ cell numbers were significantly higher in brains of treated mice than controls (TSG cells [ffLuc] only) after 5‐FC administration (Figure S8C). Furthermore, only CD‐mNSCs without tumor cells were implanted into the mouse brain, resulting in the CD8^+^ cell infiltration. This result suggested a direct immune response to the iPSC‐derived NSCs (Figure S8C). Treated mice had higher proportions of intratumoral CD8^+^ cells in CD45^+^CD3^+^ cells than controls (Figure S8D). Fewer CD163^+^ immunosuppressive cells were observed in treated mice than controls (Figure S8C). Survival was significantly prolonged in treated mice compared with controls (Figure 4d), which suggests that CD‐mNSCs with 5‐FC administration enhanced antitumor immune responses.

### 2.8 Ferroptosis induction by CD‐NSCs (ACTB)

Nonapoptotic forms of cell death including necroptosis and ferroptosis were evaluated (Figure S8E). CD‐NSC (ACTB)‐ and exogenous 5‐FU‐induced cell death (hG008 cells [ffLuc]) was rescued by N‐acetylcysteine (NAC), indicating high contribution of ferroptosis to treatment (Figure S8F,G). Conversely, TMZ‐induced cell death was partially rescued by Z‐VAD(OMe)‐FMK (Z‐VAD) (not NAC), which suggests that TMZ treatment contribution of apoptosis (Figure S8F,G).

### 2.9 Safety of CD‐NSCs (ACTB)

To evaluate the toxic effects of 5‐FC, 5‐FC was added into the medium containing iPSC‐NSCs without *yCD‐UPRT* gene or glioma cells. No killing power was observed for both iPSC‐NSCs and glioma cells (Figure S9A).

To analyze safety, CD‐NSCs (ACTB) transduced *ffLuc* ^19^ by lentiviral vector were implanted into normal brains (Figure S9B). BLI signal intensity and Venus fluorescence disappeared in mice receiving 5‐FC from Days 7 to 21 (Figure S9C–E). No differences in GFAP, NeuN, and CD31 expressions were observed at implanted sites between 5‐FC (+) and (−) brain sections (Figure S9F). Ki‐67^+^ cells were not present in brains after 5‐FC (+) administration and cleaved caspase‐3^+^ cells were present in implanted striatum (Figure S9F). There were no differences in Ki‐67^+^ and nestin^+^ endogenous mouse neural progenitor cell number in periventricular areas between 5‐FC (+) and (−) brain sections (Figure S9F). Liquid chromatography‐mass spectrometry showed 15 μM 5‐FU in 1 × 10^5^ CD‐NSCs (ACTB) supernatant after adding 5‐FC (Figure S9G,H). High local concentrations (2 μM) of 5‐FU were measured in a 7 × 7 × 7 mm site of 1 × 10^5^ CD‐NSCs (ACTB) implanted to normal brains (Figure S9G,H).

### 2.10 Biomarker to predict therapeutic response

The metabolism of 5‐FU is shown in Figure S10A. CD can convert 5‐FC to 5‐FU, which is converted to fluorouridine monophosphate (FUMP) or fluorodeoxyuridine monophosphate (FdUMP) through fluorouridine. Furthermore, FUMP is converted to fluorouridine diphosphate (FUDP) and fluorouridine triphosphate (FUTP), which inhibits RNA synthesis via the cell cycle‐independent pathway. FdUMP is converted to FdDMP and FdTMP and inhibits DNA synthesis via the cell cycle‐dependent pathway. 5‐FU has a dual mechanism of action. It directly kills the CD‐transduced cells and neighboring untransduced cancer cells through cell membranes without cell–cell junctions (bystander killing effect).^13^ The UPRT gene can directly convert 5‐FU to FUMP.^13^ hG008, GL261, and TSG showed higher sensitivity to 5‐FU than other glioma cells (Figure S10B). Significantly lower *thymidylate synthase* (*TS*) and *dihydropyrimidine dehydrogenase* (*DPD*) expressions in hG008, *GL261* and TSG than in other glioma cells were confirmed by quantitative reverse transcription PCR (Figure S10C). The dotted lines indicated that glioma cells (hG008, GL261 and TSG) with the half gene expressions of the *TS* and *DPD* compared with U87 are excellent responders to 5‐FU (Figure S10D). The present in vivo antitumor effect may be associated with not only high migratory capacity of CD‐NSCs but also the *TS* and *DPD* expressions in glioma cells.

## 3 DISCUSSION

Few studies have evaluated differences in the tumor‐trophic properties of NSCs and MSCs in the brain.^29^ We demonstrated that iPSC‐NSCs had higher tumor‐trophic migratory capacity than MSCs in the brain. RNA‐seq‐based ligand–receptor pairing analysis suggested that self‐repulsive action and pathotropism were important for iPSC‐NSC migration related to ephrin ligand/receptor signaling‐mediated repulsion in iPSC‐NSCs and CXCL12–CXCR4 interactions between GSCs and iPSC‐NSCs. EphB‐ephrinB signaling enhanced neural crest and hippocampal stem/progenitor cell migration.^30^, ^31^ CXCR4/CXCL12 signaling promoted tropism of NSCs toward glioma cells.^32^ These signaling pathways were not detected in MSCs, which suggests their role in migration differences between NSCs and MSCs. Indeed, CD‐NSCs induced strong antitumor effects even in GSC mice with diffuse invasiveness.^4^, ^33^

MSCs promoted glioma cell proliferation. Inflammatory cytokines secreted by MSCs were associated with tumor growth^34^, ^35^, ^36^ and MSCs differentiated into CAFs expressing αSMA.^37^ Furthermore, FAP^+^ fibroblasts concentrated around implanted MSCs in the brain. Therefore, MSCs might not be suitable for treating malignant tumors.

Housekeeping gene loci (*GAPDH* or *ACTB*) or safe harbor sites (*AAVS1*) were selected as efficient gene knock‐in loci in iPSCs and embryonic stem cells,^38^, ^39^, ^40^ but comparative gene expression at these target sites was not reported. *ACTB* had the highest stability under any condition.^41^ The present study suggests that *ACTB* is an appropriate locus for the stable insertion of therapeutic genes in hiPSCs.

Ferroptosis, characterized by excessive iron accumulation and lipid peroxidation,^42^, ^43^ enhanced cell immunogenicity and recruited immune cells to tumor sites.^44^ 5‐FU induced apoptosis in colorectal cancer.^45^ Here, converted 5‐FU released from CD‐NSCs induced ferroptosis (greater than exogenous 5‐FU administration) and apoptosis in GSCs, which led to antitumor immune responses. Ferroptosis requires continuous iron‐dependent reactive oxygen species formation over an extended period to trigger death.^43^ Cell‐based suicide gene therapy killed tumor over a longer period than exogenous 5‐FU administration because 5‐FU was gradually released after prodrug conversion in CD‐NSCs.

The mechanisms of resistance to 5‐FU have been previously evaluated in other malignant tumors including colorectal, breast, gastric, pancreatic, and lung cancers.^46^ TS, which is an essential enzyme for DNA de novo synthesis, and DPD, which catabolizes 5‐FU to the inactive metabolite, were measured in those studies.^46^ However, few studies focused on TS or DPD expression in glioblastomas.^47^, ^48^ The present study first demonstrated that the *TS* and *DPD* expressions predict the treatment efficacy of CD‐NSC in glioblastomas. Biomarkers to predict treatment efficacy can be utilized in the personalized medicine.

Nonlytic, amphotropic retroviral replicating vectors and immortalized human NSCs derived from human fetal brains to delivery CD^49^, ^50^, ^51^ did not affect overall survival. Virus coverage might not have encompassed the large invading glioma cell area. hiPSC‐derived NSCs might provide better therapeutic effects because of their high tumor‐trophic migratory capacity. Preclinical hiPSC‐derived NSC studies for glioblastomas are required.

Limitation of the present study was the paucity of the number of cell lines in iPSCs. The ultimate goal of the cell bank is to supply these therapeutic iPSCs of good quality at large scale. iPSCs may show different biochemical characteristics among cell lines from different donors. However, the migratory capacity of iPSCs can be quantitatively screened via our established assays including organotypic brain slice culture before implantation.

In this study, migration capacity between iPSC‐NSC was compared with adult BMSC or AMSC. However, that is inappropriate because they were derived from different tissues and different donors. In the future study, we will use iPSC‐derived NSC and the iPSC‐derived MSC,^52^, ^53^ allowing to compare migration capacity head‐to‐head with more accuracies.

Higher cell numbers and repeat cell injection may provide additional treatment effects in this strategy. Future studies with higher injected cell concentrations and repeated cell therapy are warranted to further evaluate this treatment. NSCs express various receptors for chemoattractant signals because of brain pathology. These chemoattractants are chemokines such as CXCL12 and monocyte chemoattractant protein 1, or other chemotactic proteins, such as vascular endothelial growth factor (VEGF).^7^ Therefore, further analyses are warranted to elucidate the mechanisms of migration.

## 4 CONCLUSIONS

NSCs derived from CRISRP/Cas9‐edited hiPSC have high tumor‐trophic migratory capacity and stable constitutive therapeutic transgene expression, which leads to strong antitumor effects against GSCs. The present research concept may become a platform to promote clinical studies using hiPSC.

## 5 MATERIALS AND METHODS

### 5.1 Cell culture

#### 5.1.1 Human iPSCs

1210B2‐hiPSCs^54^ were derived from human peripheral blood mononuclear cells of a healthy 29‐year‐old African/American female (Cellular Technology Limited). 1210B2‐hiPSCs (kindly provided by Shinya Yamanaka, Kyoto University, Kyoto, Japan) were cultured with a feeder‐free protocol.^12^, ^54^ Embryoid body (EB) formation and neural stem/progenitor cell generation were performed as previously described.^12^, ^55^

#### 5.1.2 Mouse iPSCs

The mouse iPS clone, 38C2,^27^ established from mouse embryonic fibroblasts, was differentiated into neurospheres via EBs in the presence of 10 M retinoic acid (Sigma‐Aldrich, Kanagawa, Japan) as described previously.^12^, ^55^

#### 5.1.3 U87 cells

A U87 human glioma cell line was obtained from the American Type Culture Collection (HTB‐14; VA, USA). Single‐cell clones stably expressing *ffLuc* gene (a Venus fluorescent protein and firefly luciferase fusion gene)^19^ were established as previously described.^4^

The U87 model is not infiltrative has an entirely abnormal and leaky vasculature and is not of glial origin.^56^ In the present study, pathotropism was evaluated using a Rose diagram map according to a previous study.^57^ The Rose diagram map can show the quantified spatial distribution of CD‐NSCs around the tumor, including both the number of CD‐NSCs in various directions and their distance from the tumor center. In this system, bulk tumor mass is used. hG008 cells diffusely infiltrated into the brain parenchyma. Therefore, U87 cells were mainly used to make a Rose diagram map in this study.

#### 5.1.4 hG008 cells

The human GSC line (hG008) was established from human glioblastoma specimens.^33^ MIF expression in hG008 cells is higher than in nonbrain tumor‐initiating cells.^33^ In tumor‐derived neurosphere culture in vitro, hG008 cells can be expanded longer than nonbrain tumor‐initiating cells. Single‐cell clones stably expressing *ffLuc* were established.^4^ We have previously reported for the first time the spatiotemporal characterization of human GSC invasion in an orthotopic xenograft mouse model using time‐lapse imaging of organotypic brain slice cultures and 3D imaging of optically cleared whole brains.^4^ GSCs in the corpus callosum migrated more rapidly and unidirectionally toward the contralateral side with pseudopod extension. These characteristics of GSC invasion shared the histological features observed in glioblastoma patients. hG008 cells (ffLuc) were cultured in ultra‐low attachment cell culture flasks (Corning, NY, USA) using the same culture conditions as that for neurospheres.^4^

#### 5.1.5 TSG cells

A mouse GSC line (TSG) was kindly provided by the Division of Gene Regulation Keio University School of Medicine and cultured with the same procedures used for hG008 cells.^58^ TSG was established by overexpressing H‐Ras (V12) in normal NSCs isolated from the subventricular zone of adult mice harboring a homozygous deletion of the Ink4a/Arf locus.^58^ Single‐cell clones stably expressing *ffLuc* were established as previously described.^4^, ^58^

#### 5.1.6 Other human and mouse glioma cell lines

Other human and mouse glioma cell lines (U251, SF126 and GL261), and human GSC line (hG020)^33^ were cultured using the same procedures used for U87 cells and hG008 cells, respectively. SF126 was obtained from the JSRB Cell Bank (IFO50286; Osaka, Japan). U251 cells were obtained from the RIKEN BRC (Ibaraki, Japan).

#### 5.1.7 MSCs and fetal NSCs

Human adipose‐derived MSCs (AMSC1 and AMSC2) were obtained from Thermo Fisher Scientific (StemPro™, R7788110) and LONZA (Tokyo, Japan; Poietics™, PT‐5006), respectively. Human bone marrow‐derived MSCs (BMSC1 and BMSC2) were obtained from LONZA (Poietics™, PT‐2501) and PromoCell (Heidelberg, Germany; C‐12974), respectively. Human fetal cortical and hippocampal NSCs (FcNSC and FhNSC) were obtained from PhoenixSongs Biologicals (CT, USA; CxB‐009 and HIP‐009). AMSCs, BMSCs, and FNSCs were transduced with the lentiviral vector CSII‐EF‐yCD‐UPRT‐IRES‐hKO1 at a multiplicity of infection (MOI) of 2. MSCs (hKO1^+^) and FNSCs (hKO1^+^) were cultured in T‐75 cell culture plastic dishes (Thermo Fisher Scientific) and laminin‐coated T‐75 cell culture plastic dish using the medium described on each product sheet, respectively.

### 5.2 Lentiviral vector‐mediated transduction

*yCD‐UPRT* fusion gene cDNA was amplified from the pDNsam‐yCD‐UPRT plasmid by polymerase chain reaction (PCR). PCR‐amplified yCD‐UPRT cDNAs were cloned into the pENTR/D‐TOPO entry vector plasmid (Thermo Fisher Scientific, MA, USA) and the final vector sequences were verified by DNA sequencing. yCD‐UPRT cDNAs were then transferred to the lentiviral vector plasmid CSII‐EF‐RfA‐IRES2‐hKO1 with Gateway LR clonase (Thermo Fisher Scientific). All plasmids are available from Addgene (https://www.addgene.org). Recombinant lentiviral vector production and titer determination were performed as described previously.^12^

### 5.3 CRISPR/Cas9‐mediated genome editing

The Cas9/sgRNA expression plasmids, pU6‐GAPDHgRNA‐Cas9, pU6‐ACTBgRNA‐Cas9, and pU6‐AAVSgRNA‐Cas9 were constructed by cloning DNA oligonucleotides coding for sgRNA targeting near the stop codon of the *GAPDH* gene, *ACTB* gene, or targeting the *AAVS1* locus into the *Bbs*I site of the pX330‐U6‐Chimeric_BB‐CBh‐hSpCas9 plasmid.^38^, ^59^, ^60^ Therapeutic NSCs (CD‐NSCs) are summarized in Table S1. To construct HR donor plasmids (Table S2), 1‐kb fragments of the left and right homology arms of the *GAPDH* gene, *ACTB* gene, and *AAVS1* locus were amplified by PCR from genomic DNA isolated from human fibroblasts, NB1RGB (RIKEN BRC), and cloned into the PrecisionX HR donor vector HR100PA‐1 (System Biosciences, CA, USA).^38^, ^59^, ^60^ Then, a polycistronic cassette containing the yCD‐UPRT and blasticidin (Bsd) or puromycin (Puro) resistance fusion gene were inserted between the left and right homology arms, resulting in the HR donor plasmids HR‐GAPDH‐2A‐yCD‐UPRT‐2A‐Bsd, HR‐GAPDH‐2A‐yCD‐UPRT‐2A‐Puro, HR‐ACTB‐2A‐yCD‐UPRT‐2A‐Bsd, HR‐AAVS1‐EF‐2A‐yCD‐UPRT‐2A‐Bsd. The HR donor plasmids of *GAPDH* and *ACTB* were designed to be in frame with the C‐terminus of *GAPDH* or *ACTB* and express the fusion proteins joined with a self‐cleaving 2A peptide sequence. The PAM sites of GAPDHgRNA and ACTBgRNA were substituted by 5′‐NTG‐3′ in the HR donor plasmids (Figure 3a). Transduced iPSCs (biallelic GAPDH) were cultured under Bsd S (2 μM) and Puro (0.2 μM) selection, and other iPSCs were cultured by Bsd S selection. All plasmids were verified by DNA sequencing.

For transfection, 1 × 10^6^ iPSCs suspended in 100 μl Opti‐MEM (Thermo Fisher Scientific) were mixed with the pU6‐GAPDHgRNA‐Cas9 plasmid (3 μg), pU6‐ACTBgRNA‐Cas9 (3 μg), or pU6‐AAVSgRNA‐Cas9 (3 μg) and the HR donor plasmid (10 μg) and then subjected to electroporation at 125 V for 5 ms using a NEPA21 electroporator (Nepa Gene, Chiba, Japan). Immediately after electroporation, cells were plated in complete medium and subjected to Bsd selection. The HR donor plasmid HR‐GAPDH‐2A‐yCD‐UPRT‐2A‐Puro (10 μg) was used for 1 × 10^6^ Bsd‐resistant iPSCs to achieve insertion into the biallelic GAPDH locus. Genomic PCR analysis was performed to verify integration.

## AUTHOR CONTRIBUTIONS

**Ryota Tamura:** Conceptualization (lead); data curation (lead); formal analysis (lead); investigation (lead); methodology (lead); resources (lead); writing – original draft (lead). **Hiroyuki Miyoshi:** Data curation (supporting); investigation (supporting); methodology (supporting); resources (supporting); supervision (supporting); validation (supporting); writing – review and editing (supporting). **Kent Imaizumi:** Data curation (supporting); investigation (supporting); validation (supporting). **Masahiro Yo:** Data curation (supporting); methodology (supporting); software (supporting); validation (supporting). **Yoshitaka Kase:** Data curation (supporting); investigation (supporting); methodology (supporting); validation (supporting). **Tsukika Sato:** Data curation (supporting); investigation (supporting); methodology (supporting); validation (supporting). **Mizuto Sato:** Data curation (supporting); investigation (supporting); methodology (supporting); validation (supporting). **Yukina Morimoto:** Data curation (supporting); investigation (supporting); validation (supporting). **Oltea Sampetrean:** Data curation (supporting); investigation (supporting); methodology (supporting); supervision (supporting); validation (supporting). **Jun Kohyama:** Investigation (supporting); methodology (supporting); supervision (supporting). **Munehisa Shinozaki:** Data curation (supporting); investigation (supporting); methodology (supporting); supervision (supporting); validation (supporting). **Atsushi Miyawaki:** Project administration (supporting); supervision (supporting). **Kazunari Yoshida:** Project administration (supporting); supervision (supporting). **Hideyuki Saya:** Project administration (supporting); supervision (supporting). **Hideyuki Okano:** Project administration (supporting); supervision (supporting); writing – review and editing (supporting). **Masahiro Toda:** Conceptualization (lead); funding acquisition (lead); supervision (lead); writing – review and editing (lead).

## FUNDING INFORMATION

This work was supported in part by grants from the Japan Society for the Promotion of Science (16K20026 to Ryota Tamura, 18K15289 to Yukina Morimoto, 17H04306 and 18K19622 to Masahiro Toda), the Japan Agency for Medical Research and Development (JP19am0401015 and JP18lm0203004 to Masahiro Toda), the Research Center Network for the Realization of Regenerative Medicine from the Japan Agency for Medical Research and Development (JP18bm0204001 to Hideyuki Okano), and the General Insurance Association of Japan. This work was partially supported by JSR Corporation as an Academic R&D project.

## CONFLICT OF INTEREST

Ryota Tamura is an inventor on patents related to genome‐edited iPSCs. Hideyuki Okano is a compensated scientific consultant of San Bio, Co., Ltd. and K Pharma Inc. and an inventor on patents related to genome‐edited iPSCs. Masahiro Toda is a founding scientist of iXgene Inc. and an inventor on patents related to genome‐edited iPSCs. The other authors declare no potential conflicts of interest.

### PEER REVIEW

The peer review history for this article is available at https://publons.com/publon/10.1002/btm2.10406.

## Supporting information


**Appendix S1** Supporting Information

**Table S4** All relevant statistical comparisons

**Movie S1** Tumor‐trophic migratory capacity of iPSC‐NSCs for diffusely infiltrative hG008 cells.
Time‐lapse imaging of slice cultures from a brain transplanted with diffusely infiltrative hG008 cells (ffLuc) together with CD‐NSCs (hKO1^+^) during a 140‐hour culture period. Images were captured every 20 min (related to Figure S7B).

**Movie S2** Tumor‐supporting effect of MSCs for U87 cells.
Time‐lapse imaging of slice cultures from a brain transplanted with hG008 cells (ffLuc) together with BMSC2 (hKO1^+^) or iPSC‐NSCs (hKO1^+^; related to Figure S3A).

**Movie S3** Anti‐tumor effect of CD‐NSC on hG008 cells.
Time‐lapse imaging of slice cultures from a brain transplanted with hG008 cells (ffLuc) together with CD‐NSCs (hKO1^+^) treated with 5‐FC or PBS (related to Figure S7B).

## Data Availability

The RNA‐seq data have been deposited in the NCBI Gene Expression Omnibus (GEO) under accession number GSE150470. All other data in this article are available from the corresponding author upon reasonable request.
