# Supplementary material for: Gene therapy using genome‐edited iPS cells for targeting malignant glioma
Source: Bioeng Transl Med. 2022 Sep 10;8(5):e10406. doi: 10.1002/btm2.10406 (PMC10487333; doi:10.1002/btm2.10406)
Supplement: Supplementary file 1 — Appendix S1 Supporting Information [file BTM2-8-e10406-s004.docx]

**Supporting Information**

**Gene therapy using genome-edited iPS cells for targeting malignant glioma**

**Authors:**

Ryota Tamura, M.D., Ph.D.,^1^ Hiroyuki Miyoshi, Ph.D.,^2†^ Kent Imaizumi, M.D.,^2^ Masahiro Yo, Ph.D.,^3^ Yoshitaka Kase, M.D., Ph.D.,^2,5^ Tsukika Sato,^2^ Mizuto Sato, M.D.,^1^ Yukina Morimoto, M.D.,^1^ Oltea Sampetrean, M.D., Ph.D.,^4^ Jun Kohyama, M.D., Ph.D.,^2^ Munehisa Shinozaki, M.D., Ph.D.,^2^ Atsushi Miyawaki, M.D., Ph.D.,^3^ Kazunari Yoshida, M.D., Ph.D.,^1^ Hideyuki Saya, M.D., Ph.D.,^4^ Hideyuki Okano, M.D., Ph.D.,^2^ and Masahiro Toda, M.D., Ph.D.^1*^

**Affiliations:**

^1^Department of Neurosurgery, ^2^Department of Physiology, ^4^Division of Gene Regulation, Institute for Advanced Medical Research, Keio University School of Medicine, 35 Shinanomachi, Shinjuku-ku, Tokyo, 160-8582, Japan

^3^Laboratory for Cell Function and Dynamics, RIKEN Center for Brain Science, 2-1, Hirosawa, Wako, Saitama, 351-0106, Japan

^5^Department of Geriatric Medicine, Graduate School of Medicine, The University of Tokyo, Bunkyo-ku, Tokyo 113-8655, Japan.

***Correspondence should be addressed to** Masahiro Toda, Department of Neurosurgery, Keio University School of Medicine, 35 Shinanomachi, Shinjuku-ku, Tokyo, 160-8582, Japan. E-mail: [todam@keio.jp](mailto:todam@keio.jp); Telephone: +81-3-3353-1211; Fax: ＋81-3-5843-6167.

**Table of contents:**

**1. Supplementary Methods**

**2. Supplementary Figures: Figure S1-S10**

**3. Supplementary Tables: Table S1-S4**

**4. Supplementary Movies: Movie S1-S3**

**5. References**

**1. Supplementary Methods**

**1.1. Cell viability assay**

Cell viability assay was performed to evaluate the sensitivity of each CD-NSC to 5-FC (Sigma-Aldrich, MO, USA), and the sensitivity of each tumor cell to 5-FU (Sigma-Aldrich) and TMZ (LKT laboratories, MN, USA), which is the standard chemotherapy for glioblastoma [61]. The cell viability assay was performed 3 days after adding each drug using the Cell Counting Kit-8 (CCK-8; Dojindo Molecular Technologies, Kumamoto, Japan) as previously described [12]. Each experiment was performed in triplicate.

**1.2. Transwell assay**

For separate culture, AMSCs, BMSCs, FcNSC, and iPS-NSC were plated onto BD Falcon^®^ cell culture inserts with 0.4μm pores in a permeable membrane (Corning) at a density of 5×10^4^ cells per insert. U87 cells (ffLuc) were seeded into a Falcon 6-Well culture companion plate (BD Biosciences, CA, USA) at a density of 5 ×10^4^ cells per well and incubated for 5 days. The culture medium used depended on cells in the inserts. After incubation, the cell growth assay was performed for U87 cells (ffLuc). U87 cells (ffLuc) cultured with inserts alone (without cells) were used as controls.

**1.3. Cytokine arrays and image analysis**

MSCs, FcNSCs, or iPS-NSCs were co‐cultured with U87 cells (ffLuc) in a Falcon 6-Well culture companion plate (BD Biosciences) in the presence of culture medium (1:1 ratio, 5×10^4^ cells per well). Human cytokine profiling was performed using a Proteome Profiler Human Cytokine Array Kit ARY005B (R&D Systems, MN, USA), which detects 36 human soluble cytokines. Following incubation for 4 days, 1.5mL of culture supernatant was collected. Culture supernatant from each stem cell was used as the control. Following incubation with a detection antibody cocktail, immunoblots on the membrane were developed with a chemiluminescent substrate reagent kit and visualized using a LAS-4000mini luminescent image analyser (Fujifilm, Tokyo, Japan). Cytokine array images were analysed using ImaGene 8.0 software (BioDiscovery, Inc., CA, USA). The mean signal intensity of the cytokine/chemokine spots was divided by the mean signal intensity of the reference spots for each membrane.

**1.4. Analysis of neurosphere proliferation**

To assess the proliferation rate of each NSC derived from hiPSC *in vitro*, neurospheres were dissociated into a 6-well culture plate (1×10^5^/mL). Following incubation for 2 days, the size and number of neurospheres were analysed using an All-In-One Fluorescence Microscope BZ-H3A (Keyence, Osaka, Japan), as previously described [62]. The frequency of neurospheres larger than 150 μm was calculated as the percentage of total neurospheres.

**1.5. Western blotting**

The CD protein expression was measured in each CD-NSC by western blotting, as previously described [62]. β-actin was used to normalize the protein expressions. Protein was isolated using the miRNeasy Serum/Plasma kit (QIAGEN, Tokyo, Japan) with QIAcube (QIAGEN) automation. Anti-yCD antibody (1:50, Bio-Rad, CA, USA; 2485-4906), anti-β-actin antibody (1:500, Sigma-Aldrich; A1978), anti-GAPDH antibody (1:1000, Cell Signaling Technology, Tokyo, Japan; D16H11) were used as primary antibodies, and the appropriate HRP-conjugated secondary antibodies were used (1:3000, Jackson ImmunoResearch, PA, USA).

**1.6. Quantitative reverse transcription PCR (RT-qPCR)**

Total RNA was isolated using the miRNeasy Serum/Plasma kit (QIAGEN) with QIAcube (QIAGEN) automation. RNA concentration was measured using the Nanodrop 8000 (Thermo Fisher Scientific). Complementary DNA was reverse transcribed using a ReverTraAce qPCR RT kit (TOYOBO, Osaka, Japan). qPCR was performed using SYBR® Premix Ex Taq™ II (Takara Bio, Inc., Shiga, Japan) and a ViiA 7 Real-Time PCR System (Thermo Fisher Scientific) according to the manufacturer’s protocol. Fold changes were calculated using the ΔΔCt comparative quantification method. ΔCT was determined as the difference between human β-actin and the tested gene. The primer sequences are listed in Table S3.

**1.7. Immunohistochemistry and cytochemical analyses**

Standard Immunohistochemistry and cytochemical analyses were performed. For immunohistochemistry, 20-µm tissue sections stored in sterile antifreeze solution were stained. The primary antibody were anti-mKO2 (hKO1) antibody (diluted 1:500, rabbit IgG1; MBL, Nagoya, Japan; PM051M), anti-GFAP antibody (diluted 1:200, rat IgG2a; Thermo Fisher Scientific; 2.2B10), anti-NeuN antibody (diluted 1:200, mouse IgG1; Merck, Tokyo, Japan ; A60), anti-nestin antibody (diluted 1:150, rat IgG1; Gene Tex, CA, USA ; 4D4), anti-CD31 antibody (diluted 1:150, rat IgG2a; 550274, BD Biosciences; MEC 13.3), anti-αSMA antibody (diluted 1:200, mouse IgG2a; Thermo Fisher Scientific; 1A4), anti-FAP antibody (diluted 1:100, sheep IgG1; R&D Systems, MN, USA; AF3715), anti-Cleaved caspase 3 antibody (diluted 1:200, rabbit IgG1; Cell Signaling Technology; Asp175), anti-STEM121 antibody (diluted 1:500, mouse IgG1; Cellartis–Takara Bio, Inc.; Y40410), anti-CD8 antibody (diluted 1:400, rabbit IgG1; Cell Signaling Technology; D4W2Z), anti-CD163 antibody (diluted 1:500, rabbit IgG1, Abcam, Cambridge, UK; EPR19518), anti-GAPDH antibody (diluted 1:200, rabbit IgG1; Cell Signaling Technology; D16H11), and anti-CD antibody (diluted 1:200, rabbit IgG1, Bioss antibodies, MA, USA; bs-2950R). The primary antibodies were detected using the appropriate Alexa Fluor conjugated secondary antibodies (diluted 1:500; Thermo Fisher Scientific). Samples were mounted with VECTASHIELD Antifade Mounting Medium containing DAPI (Vector Laboratories, CA, USA), and examined by All-In-One Fluorescence Microscope (BZ-H3A, Keyence), and confocal laser scanning microscope LSM700 (Carl Zeiss, Oberkochen, Germany).

**1.8. 5-ethynyl-2′-deoxyuridine (EdU) staining**

EdU (Cell-Light EdU Cell Proliferation Detection kit; Guangzhou RiboBio Co., Ltd., Guangzhou, China) was used to evaluate the proliferation of each CD-NSC as previously described [62]. A confocal laser scanning microscope LSM700 (Carl Zeiss) or an All-In-One Fluorescence Microscope BZ-H3A (Keyence) was used for quantification. The frequency of neurospheres containing immunopositive cells was calculated as the percentage of total neurospheres (n = 10 independent cultures; at least 1,500 cells were analysed)

**1.9. FACS**

Primary tumor cell suspensions were stained with anti-mouse CD45 (BD Bioscience; 30-F11), anti-mouse CD3 (BioLegend, CA, USA; 145-2c-11), anti-mouse CD4 (BioLegend; RM4-5), and anti-mouse CD8 (BioLegend; 53-6.7). An isotype control sample for each condition was used to exclude the autofluorescence background. Lymphocytes isolated from tumor tissues were gated on CD3^+^ cellular elements and helper or cytotoxic T lymphocytes were distinguished based on the sole expression of CD4 or CD8 marker, respectively. Analyses were performed using a Gallios Flow Cytometer (Beckman Coulter, CA, USA) by the same operator. Data were analysed using Kaluza Analyse software (Beckman Coulter).

**1.10. Liquid chromatography-mass spectrometry (LC-MS)**

5-FC (15µM) wad added into the medium containing CD-NSCs (ACTB) at a density of 1×10^5^ cells per well (1.0 mL). Following incubation for 4 days, culture supernatant was collected and 1×10^5^ CD-NSCs (ACTB) / 2 μl were transplanted to a point 3mm deeper from the brain surface through a burr hole 2 mm lateral to the bregma as previously described [4]. Mice received 5-FC (500mg/kg, intraperitoneally) once daily from Day 2 to 16. Brain tissues were obtained from mice without perfusion and cut into three parts of 100mg each (See Figure S8F). LC-MS were performed for these samples by Kazusa DNA Res. Inst (Chiba, Japan).

**1.11. RNA-seq**

For RNA-seq, we extracted RNA from iPSC-NSCs, iPSC-NSCs with yCD-UPRT (CD-NSCs [ACTB]), FNSCs, AMSCs and BMSCs using the miRNeasy Serum/Plasma kit (QIAGEN) with QIAcube (QIAGEN) automation. The indexed cDNA libraries were prepared using the TruSeq stranded mRNA Library Preparation kit (Illumina), and were sequenced using a NovaSeq6000 (Illumina) to obtain 100-bp paired-end reads. Published RNA-seq data of resected samples of glioblastoma and parental intact brain tissues [63] were downloaded via NCBI Short Read Archive (SRR359290 and SRR359291). Raw FASTQ files were trimmed for adapters by Cutadapt [64] and Salmon [65] was used to generate the TPM and estimated counts using the transcript index from GRCh38 (Gencode v33). We identified differentially expressed genes using the DESeq2 [66] suite of bioinformatics tools with a cutoff of 0.001 for Benjamini–Hochberg adjusted p-values and a cutoff of 10 for the fold-change ratio. PCA analysis was performed using vst transformation of estimated counts. Enrichment analyses using gene ontology (GO) and MsigDB genesets were performed using clusterProfiler [67]. Heatmaps of gene expression were drawn on the log2(TPM + 1) value or the row-wise z-value of log2(TPM + 1) for each gene. The RNA-seq dataset has been deposited in the NCBI Gene Expression Omnibus and is accessible through GEO series accession number GSE150470.

*Reanalysis of published RNA-seq data*

Published RNA-seq data of glioblastoma [23-25] were reanalyzed. We obtained processed datasets including TPM values or raw count values from the NCBI Gene Expression Omnibus. TPM values were calculated as the average of each glioblastoma sample.

*Ligand-receptor pairing analysis*

Analysis of receptor–ligand pairings was performed as previously reported [68]. Overall, 2,552 curated receptor–ligand pairs were selected [69] and an expression cutoff of log2(TPM + 1) = 3 was used. Receptor–ligand pairs were classified as matched pairs if both the ligand and receptor were expressed.

**1.12. Whole-genome sequencing**

The degree of genome-wide off-target mutagenesis in CD-iPSCs was assessed. Genomic DNA was extracted using a NucleoSpin Tissue kit (Takara Bio, Inc.) according to the user’s manual. After the preparation of a DNA library with the MGIEasy FS DNA Library Prep Set (MGI Tech), genomic sequence analysis was performed by DNBSEQG400 (2 × 100 bp). Raw FASTQ files were trimmed for adapters by Cutadapt, and raw reads from each sample were mapped or aligned to the human reference genome (GRCh38) by BWA. SNPs and indels were identified using SAMtools and filtered using bcftools. The gRNA-A1 was aligned to GRCh38 using Cas-OFFinder allowing up to 5 mismatches to predict potential off-target sites at the whole-genome. The all mutations of hiPSC-CD detected in the potential 20 bp off-target sites were compared to that of hiPS-WT.

**1.13. Evaluation of apoptosis, necroptosis, and ferroptosis**

hG008 (ffLuc) were cultured for 48 hours in the presence of N-acetylcysteine (NAC, 3 mM), Z-VAD(OMe)-FMK (Z-VAD, 30 µM), ferrostatin-1 (Fer, 2 µM), or necrostatin-1 (Nec-1, 30 µM), and then TMZ (200µM) or 5-FU (50µM) were added. To evaluate the type of cell death mediated by CD-NSCs (ACTB), hG008 (ffLuc) and CD-NSCs (ACTB) were co-cultured for 48h in the presence of an inhibitor and then 5-FC (15µM) was added. Cell viability was evaluated using a CCK-8 assay.

**1.14. Organotypic brain slice culture and image analysis**

Organotypic brain slice culture was performed as previously described [4,12]. Female BALB/c nude mice (6 weeks old; 20g; Sankyo Labo Service Corporation. Inc., Tokyo, Japan) were anesthetized with equithesin and placed in a stereotaxic apparatus (Narishige Scientific Instrument Lab., Tokyo, Japan). iPSC-NSCs, FcNSCs, AMSCs and BMSCs were transduced with the lentiviral vector CSII-EF-yCD-UPRT-IRES-hKO1 at a multiplicity of infection of 2. To evaluate the migration capacity of each stem cell, 1×10^5^ of U87 cells (ffLuc) / 2μl were transplanted using a 10-μl Hamilton syringe to the striatum of a mouse (Day 0). On Day 5, 5×10^5^ of iPSC-NSCs, FcNSCs, AMSCs, BMSCs with hKO1 fluorescence were transplanted to a point 2mm deep from the brain surface (1mm above the U87 [ffLuc]-derived tumor). Time-lapse imaging of slice cultures was performed for 3 days starting on Day 7. To verify the association between the CXCL12/CXCR4 pathway and migration, brain slices were treated with a CXCR4 antagonist (AMD3465) for 3 days. The concentrations of AMD3465 were 1 µM. To verify the association between the EphB-ephrinB pathway and migration, brain slices were treated with a specific EphB4 inhibitor (NVP-BHG712) for 3 days. The concentrations of NVP-BHG712 was 33 nM. The ligand-receptor pairing analysis demonstrated that there is notable change in EphB3 and EphB4 expression between NSCs and MSCs. Previous study has shown that EphB4 is involved in neuronal differentiation, thus EphB4 is predicted to be expressed in almost all NSC [70]. Therefore, a specific EphB4 inhibitor (NVP-BHG712) was selected in this study.

To evaluate the anti-tumor effect, 5×10^5^ of hG008 cells (ffLuc) / 2μl were transplanted to the striatum. On Day 45, 5×10^5^ hiPSC-NSCs with yCD-UPRT were transplanted to a point 2 or 3mm deeper from the brain surface. On Day 47, time-lapse imaging of slice cultures was performed for 6 days. On Day 49, the medium was replaced and 5-FC (250µM) was added.

**1.15. Brain Clearing**

Female BALB/c nude mice (Sankyo Labo Service Corporation. Inc.) were used. First, 5×10^5^ of hG008 (ffLuc) / 2μl were transplanted to the striatum. On Day 35, 5×10^5^ of CD-NSCs transduced with the lentiviral vector CSII-EF-yCD-UPRT-IRES-hKO1 were transplanted to a point 3mm deep from the surface of the brain with or without hG008 cells (ffLuc). On Day 50, the brain tissues were obtained. PASSIVE CLARITY-brain clearing was performed as previously described [4,71]. Three-mm thick coronal sections, except cerebellum and olfactory bulb, were cut. Imaging was performed by multi-photon microscopy (FLUOVIEW FVMPE-RS; Olympus, Tokyo, Japan) and 3D images were reconstructed using FV31S-SW software with a maximum intensity projection algorithm (Olympus).

**1.16. *In vivo* assessment in an orthotopic mouse model**

NSCs differentiated from 1210B2 hiPSCs were dissociated into single cells and then transduced with the lentiviral vector CSII-EF-yCD-UPRT-IRES-hKO1 (CD-NSC [Lenti]). yCD-UPRT was inserted into the monoallelic *GAPDH*, biallelic *GAPDH*, *AAVS1*, or *ACTB* loci in 1210B2 hiPSCs, and subsequently differentiated into NSCs (CD-NSCs [mGAPDH, bGAPDH, AAVS, and ACTB], respectively). CD-NSC (bGAPDH) was established to increase yCD-UPRT gene expression because CD-NSC (mGAPDH) did not show marked sensitivity to 5-FC. CD-NSCs (ACTB) were used to evaluate anti-tumor effects for human GSC (hG008) orthotopic xenograft mouse models. NSCs differentiated from 38C2 mouse iPSCs were dissociated into single cells and then transduced with the lentiviral vector CSII-EF-yCD-UPRT-IRES-hKO1 (CD-mNSC) to evaluate T cell-mediated anti-tumor immune responses in an immunocompetent mouse GSC (TSG) model. Histopathological analysis was performed as previously described [12]. All experiments were performed in accordance with the Guidelines for the Care and Use of Laboratory Animals of Keio University (Approval number: 14057) and the Guide for the Care and Use of Laboratory Animals (NIH).

*Evaluation of the anti-tumor effects of CD-NSCs on human GSC (hG008) orthotopic xenograft mouse models*

Female BALB/c nude mice were used. To evaluate the *in vivo* anti-tumor effects of CD-NSCs (ACTB), 5×10^5^ hG008 cells (ffLuc) / 2μl was transplanted to a point 3mm deep from the brain surface through a burr hole 2 mm lateral to the bregma as previously described [12]. On Day 35, 5×10^5^ of CD-NSCs (ACTB) were transplanted to the same region. To evaluate the therapeutic efficacy, BLI to monitor hG008 cell (ffLuc) growth *in vivo* and Kaplan-Meier survival analysis were performed. In each experiment, three groups were tested: control mice transplanted with hG008 cells (ffLuc) followed by the administration of 5-FC or transplanted with hG008 cells (ffLuc) and CD-NSCs (ACTB) followed by the administration of PBS, and treatment mice were transplanted with hG008 cells (ffLuc) and CD-NSCs (ACTB) followed by the administration of 5-FC. Treated mice received 5-FC (500 mg/kg, intraperitoneally) once daily from Day 42 to Day 56. Tumor volume was measured after sacrifice by decapitation at Day 49 and 56.

*Evaluation of the anti-tumor effects of CD-NSCs in an immunocompetent mouse GSC (TSG) model*

Female C57BL/6 mice (6 weeks old; 20g; Sankyo Labo Service Corporation. Inc.) received 5×10^5^ TSG cells (ffLuc) / 2 μl transplanted to the right striatum. On day 5, 5×10^5^ of CD-mNSCs were transplanted to the same region. In each experiment, three groups were tested: control mice transplanted with TSG cells (ffLuc) followed by the administration of 5-FC or transplanted with TSG cells (ffLuc) and CD-mNSCs followed by the administration of PBS, and treatment mice were transplanted with TSG cells (ffLuc) and CD-mNSCs followed by the administration of 5-FC. Treated mice received 5-FC (500 mg/kg, intraperitoneally) once daily from Day 7 to Day 21. Tumor volume was measured after sacrifice by decapitation at Day 14 and 21. Furthermore, only CD-mNSCs without tumor cells were implanted into the right striatum to evaluate direct immune responses to the iPSC-derived NSCs. All other experiments were performed with the same protocols used for U87 cells. Brain tissues were obtained on Day 21 for tumor volume and FACS analysis.

*IVIS*

A Xenogen-IVIS 100 imaging system (version 4.3.1; PerkinElmer, MA, USA) was used for *in vivo* BLI as previously described [12]. Images were quantiﬁed as photons per second for TSG cells (ffLuc) and per minute for hG008 cells (ffLuc) and CD-NSCs (ffLuc).

*Safety Evaluation*

CD-NSCs (ACTB) were transduced with the lentiviral vector CSII-EF-*ffLuc*. CD-NSCs (ACTB) stably expressing *ffLuc* were established, then 5×10^5^ of CD-NSCs (ACTB; ffLuc) were transplanted into the striatum of normal brains as described above [12]. To evaluate safety, BLI was performed to monitor CD-NSC (ACTB; ffLuc) growth *in vivo*. Mice received 5-FC (500mg/kg, intraperitoneally) or PBS once daily from Day 7 to Day 21. Histological analysis was performed after sacrifice by decapitation at Day 60.

To evaluate the toxic effects of 5-FC, 5-FC was added into the medium containing iPSC-NSCs without *yCD-UPRT* gene or glioma cells. Cell viability assay was performed, as described above.

*BLI to monitor the migration of CD-NSCs*

U87 cells (1×10^4^) transduced with the lentiviral vector containing the *hKO1* gene were transplanted into the left striatum (Day 0). Then, 5×10^5^ CD-NSCs (ACTB; ffLuc) were transplanted into the same region or the contralateral side (right striatum). Migration activity *in vivo* was analysed using BLI to monitor CD-NSCs (ACTB; ffLuc). Histological analysis was performed after sacrifice by decapitation at Day 35.

**1.17. Statistical analysis**

Survival curves were analysed by a log-rank test based on the Kaplan–Meier test. Depending on the type of experiment, data were tested using Student’s *t*-test, and one-way ANOVA with post-hoc test. All relevant comparisons were shown in the Table S4. Analyses were performed with IBM SPSS statistics (IBM Corp., NY, USA). A *P*-value of <0.05 was considered statistically signiﬁcant. All experiments were independently repeated twice.

**2. Supplementary Figures**


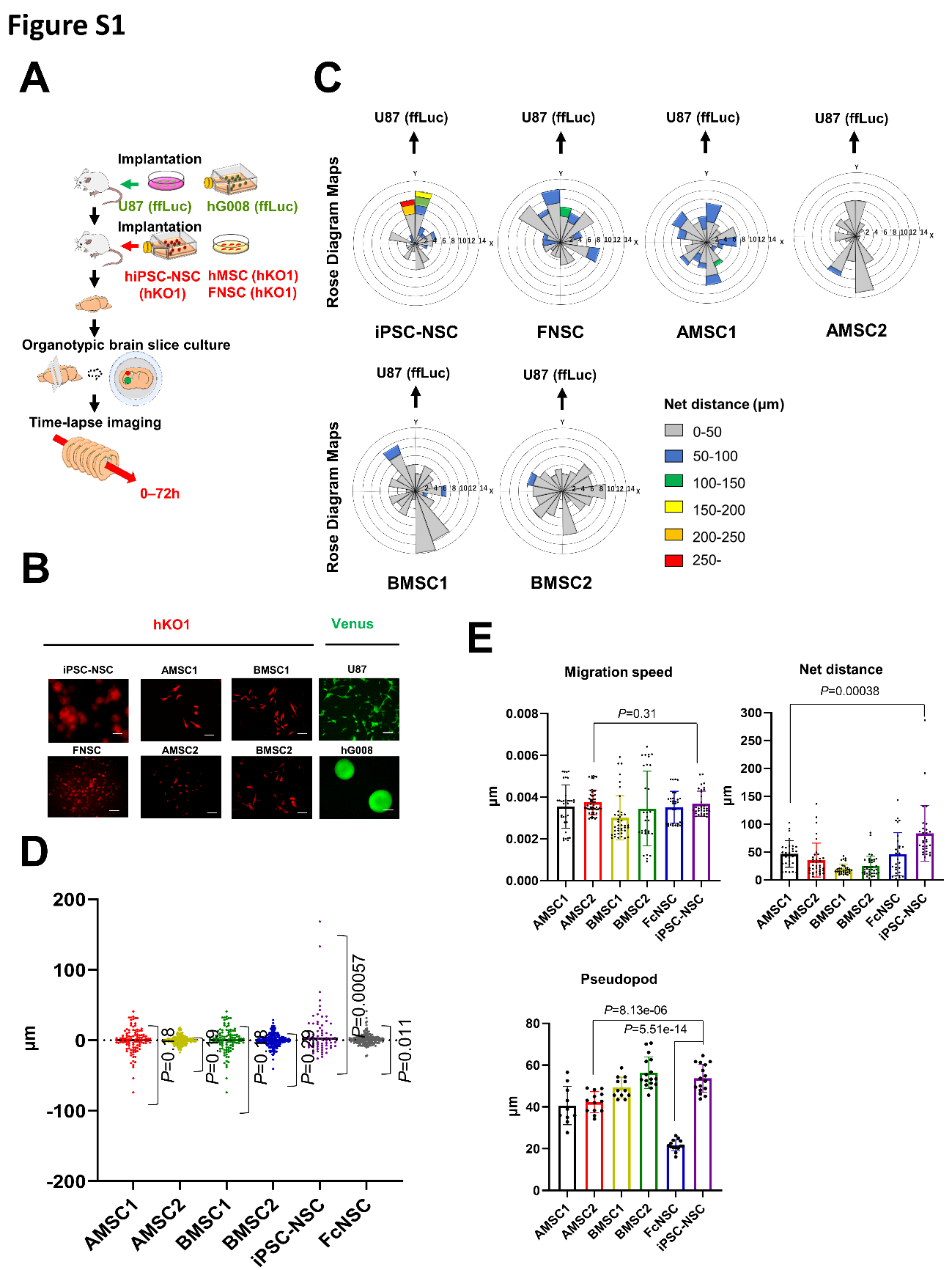
 **Figure S1.** **Migratory capacity of NSCs and MSCs.** (A) Schematic overview of the experimental procedure using an organotypic brain slice culture. (B) Rose diagram map displays the distribution of implanted cells around the circle and shows the final cell distribution with respect to their initial position in a 360˚ circle divided by 20˚. The Y-axis is the direction toward the mass of U87 cells (ffLuc). The radii of wedges indicate cell numbers and the innermost wedge indicates 1 with an increment of 1 for the outer wedges. Individual cells are classified according to their net distance of migration by the indicated colours. (C) *in vitro* growth of NSCs and MSCs expressing hKO1, and U87 cells and hG008 cells expressing Venus. Scale bars, 100 mm. (D) Y-axis positive and negative components of net distance are shown for each type of stem cell. (E) Quantitative analysis of migration speed, net distance, and pseudopods for each type of stem cell in slice cultures from brains transplanted with the indicated cells. Migration speed was calculated during 72-hour culture. Migration distance was measured as the net distance between the start and end points during 72- hour culture. The mean (bar) ± SD (error bars) is shown.


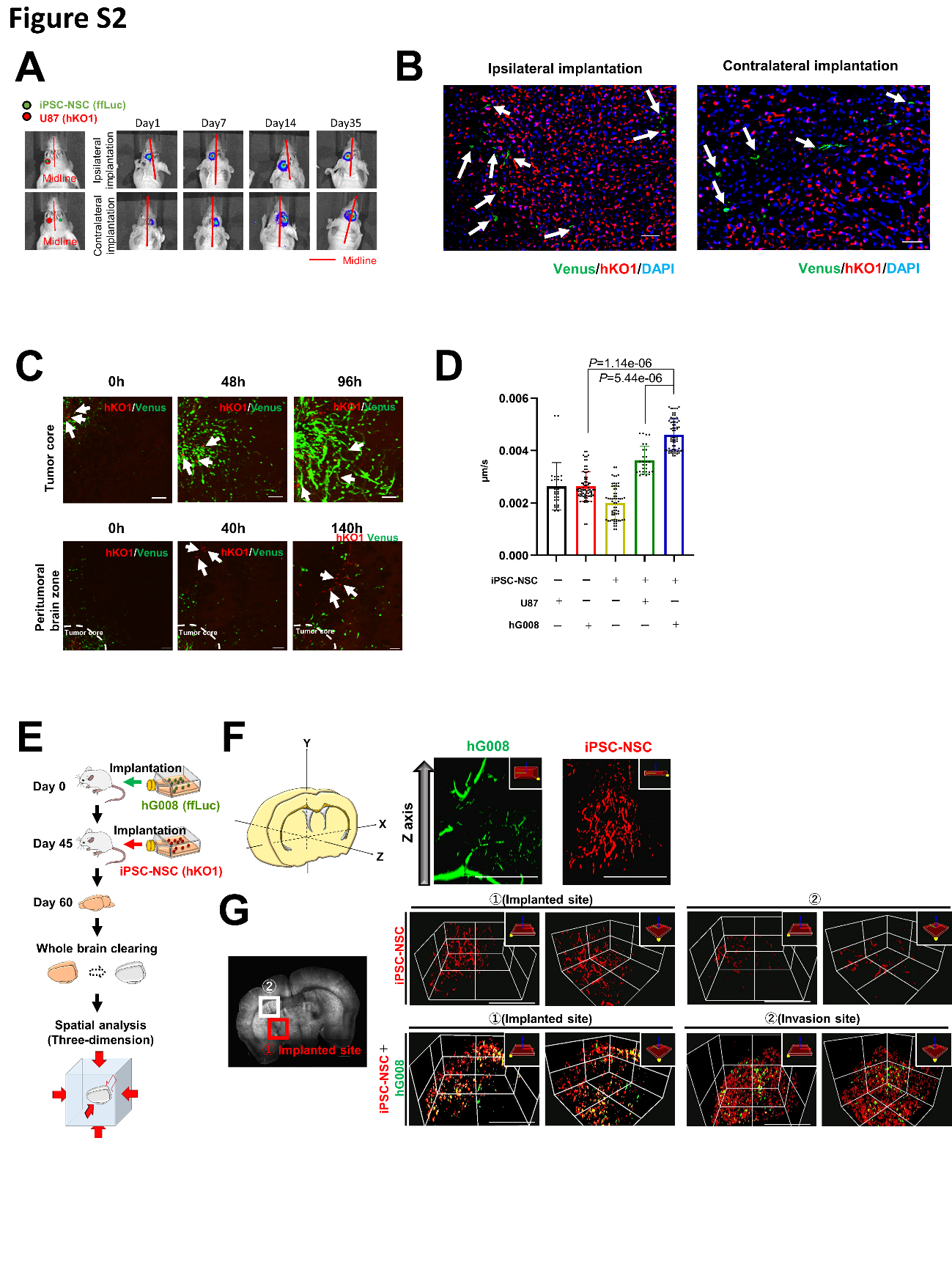


**Figure S2.** **Migratory capacity of iPSC-NSC.**

(A) Panels show a time series for hiPSC-NSCs transfected with the *ffLuc* gene. hiPSC -NSCs (ffLuc) were implanted into the contralateral (right) or ipsilateral side (left) of U87 (hKO1^+^)-derived tumors (left). Photon emission imaging of Luc expression at days 1, 7, 14, and 35. Migration towards the tumor is evident from day 14. The coloured scale bar represents BLI radiance intensity in photons/minute/cm^2^/steradian. Red line, midline. (B) Thirty-five days after brains were transplanted with U87 cells (hKO1^+^; left) and hiPSC-NSCs (ffLuc), they were stained with anti-hKO1 antibody and anti-GFP antibody. hiPSC-NSCs (ffLuc) are shown in the tumor on the contralateral side. Scale bar, 100 μm. (C) Snapshot fluorescence images of slice cultures from brains transplanted with diffusely infiltrative hG008 cells (ffLuc) and hiPSC-NSCs (hKO1^+^) at the indicated timepoints. Scale bar, 100 μm. White arrow, hiPSC-NSCs (hKO1^+^). See Additional file 7: Movie S1. (D) Slice cultures from brains transplanted with hiPSC-NSCs (hKO1^+^) and U87 cells (ffLuc), or hiPSC-NSCs (hKO1^+^) and hG008 cells (ffLuc). Trajectories of migrating hiPSC-NSCs (hKO1^+^) during the first 48 hours in culture are evaluated. Quantitative analysis of migration speed of U87 cells (ffLuc), hG008 cells (ffLuc) and hiPSC-NSCs (hKO1^+^) in slice cultures from brains transplanted with the indicated cells. The mean (bar) ± SD (error bars) is shown. (E) Schematic overview of the experimental procedure for brain clearing. hiPSC-NSCs with hKO1 fluorescence were implanted into the same region as hG008 cells (ffLuc). Brain tissues of mice were cleared 15 days after the implantation of hiPSC-NSCs (hKO1^+^) and 3D fluorescence images were reconstructed. Brain tissues with hiPSC-NSCs (hKO1^+^) only were used as controls. (F) Representative fluorescence images of the striatum. Pseudopods of hiPSC-NSCs (hKO1^+^) and hG008 cells (ffLuc) are shown along the z-axis at the moment of migration into the striatum. Scale bar, 250µm. (G) Representative fluorescence images of the invasion site (② white square) above the implanted site (① red square). In brains implanted with hG008 cells (ffLuc), hiPSC-NSCs (hKO1^+^) concentrated around the invasion site of hG008 cells (ffLuc). Conversely, in brains without hG008 cells (ffLuc), most of hiPSC-NSCs (hKO1^+^) were observed around implanted sites. Scale bar, 250µm.


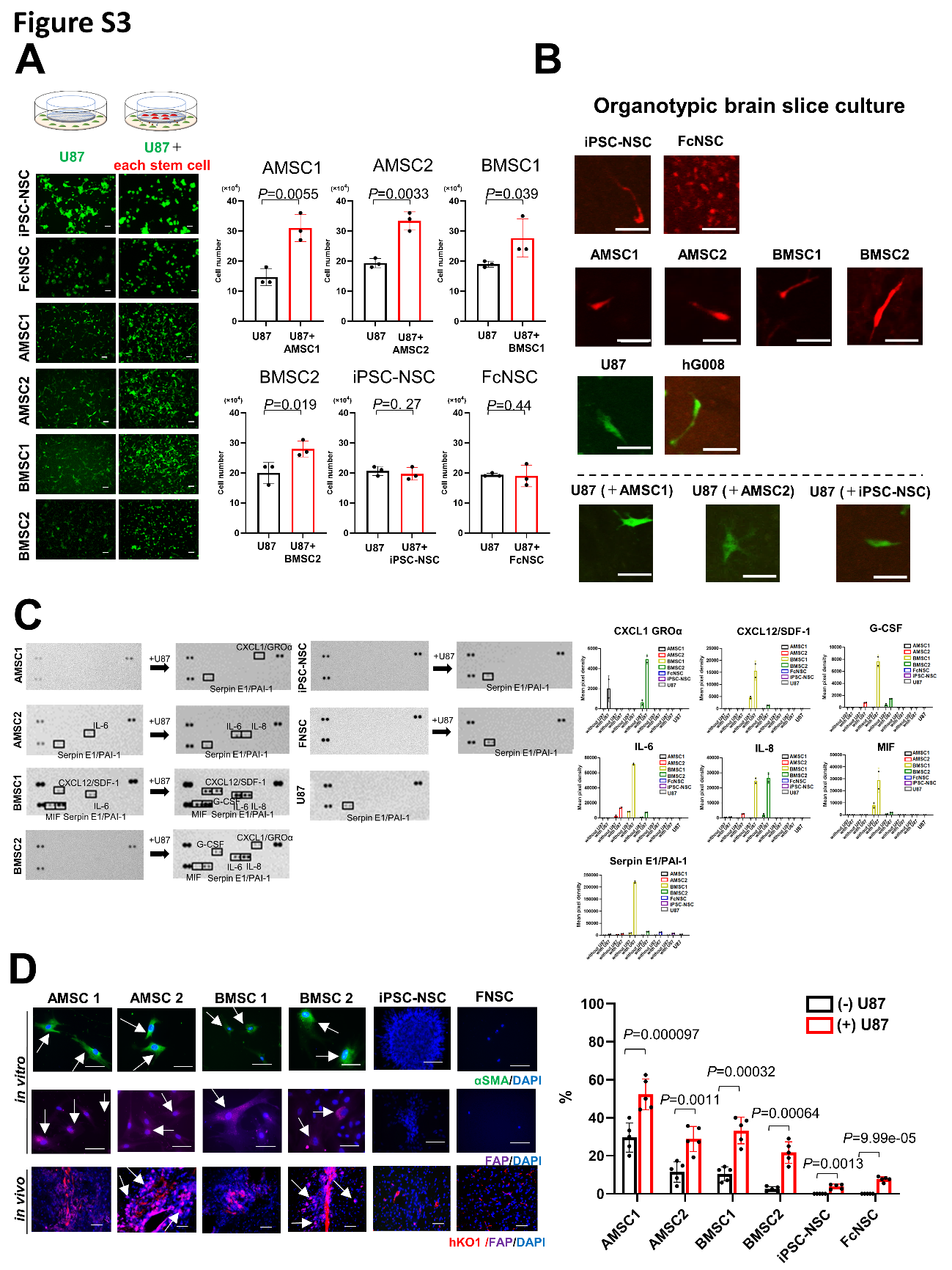


**Figure S3.** **Tumor-supporting effect of MSCs.**

(A) A co-culture system was developed with each type of stem cell in the upper compartment and U87 cells (ffLuc) in the bottom compartment of a transwell chamber. The mean (bar) ± SD (error bars) is shown. U87 cells (ffLuc) cultured with inserts alone (without cells) were used as controls. Scale bar, 100 μm. See Additional file 8: Movie S2. (B) Pseudopods of each type of cell line in organotypic brain slice culture are shown. hiPSC-NSCs (hKO1^＋^), BMSCs (hKO1^＋^), and hG008 cells (ffLuc) exhibit a longer pseudopod than other cell lines. U87 cells (ffLuc) exhibit multipolar pseudopods in the presence of MSCs, in contrast to the monopolar pseudopod of original U87 cells (ffLuc). Scale bar, 100 μm. (C) Secreted cytokines were measured by a human cytokine array panel. Culture supernatant from each type of stem cell alone was used as a control. Mean pixel density of CXCL1/GROα, G-CSF, MIF, CXCL12/SDF-1, IL-6, IL-8 and Serpin E1/PAI-1 are shown. (D) Immunocytochemical staining of αSMA and FAP on NSCs and MSCs. Brain sections from the indicated mice were stained with anti-FAP antibody and DAPI. hiPSC-NSCs, FNSCs and MSCs were detected by hKO1 staining. The percentage of FAP^+^ cells when each type of stem cell was co-cultured with/without U87 cells (ffLuc). The mean (bar) ± SD (error bars) is shown. Scale bar, 100 μm. White arrow, αSMA or FAP^+^ cells.


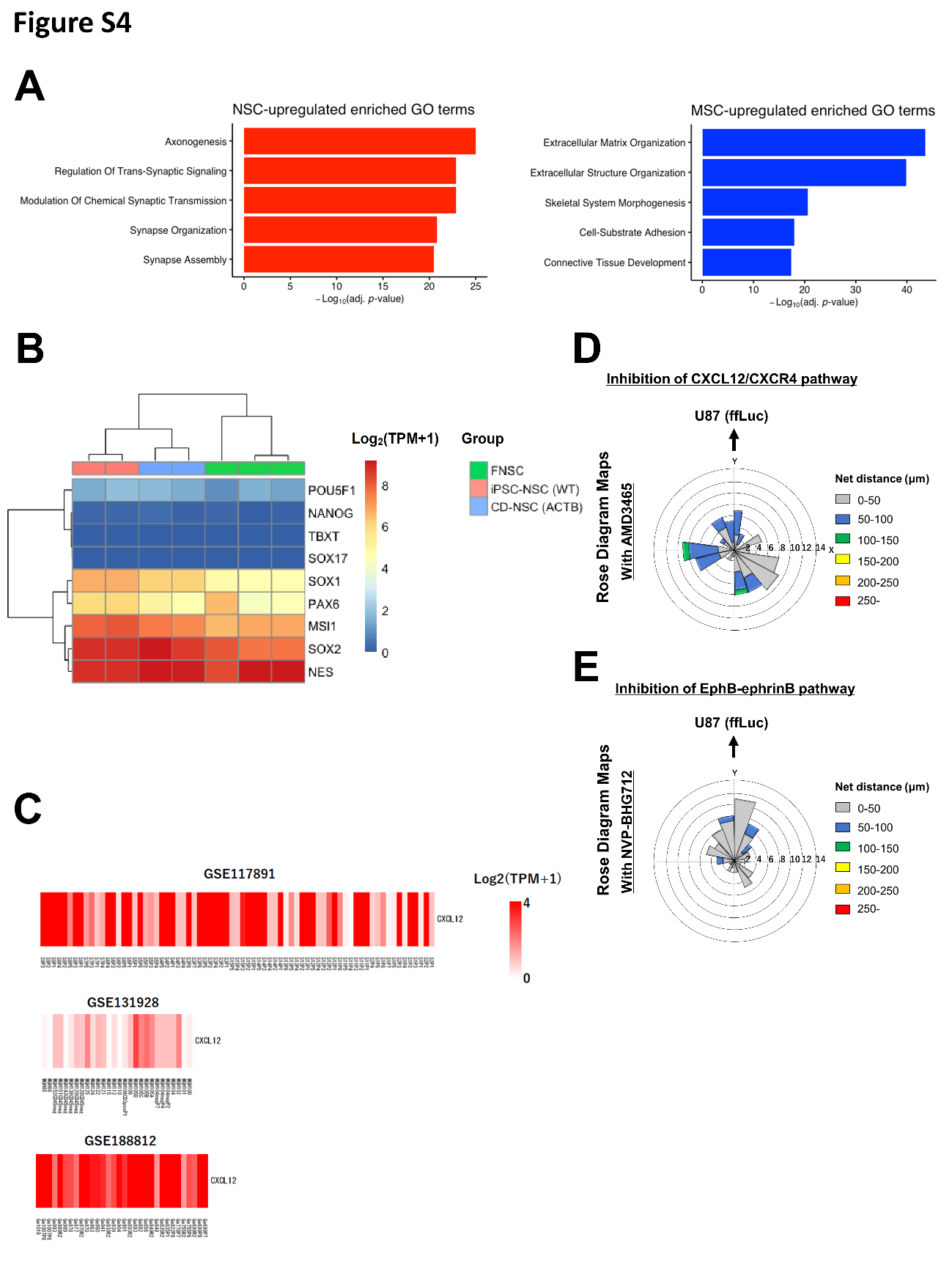


**Figure S4.** **RNA-seq data analysis.**

(A) GO analysis of NSC- and MSC- upregulated genes. NSC-upregulated genes were enriched with “axon” and “synapse functions”. Conversely, MSC-upregulated genes were associated with “extracellular components”. (B) Heat-map of NSC-related marker expression. (C) Reanalysis of published RNA-seq data: GSE117891, GSE131928 and GSE188812. Heatmap showing the expression of CXCL12 in each resected glioblastoma sample. (D) The migration assay was performed using a CXCR4 antagonist. Rose diagram map demonstrated that CXCR4 blocked CXCL12-mediated pathotropism of NSC toward glioma cells. hiPSC-NSCs tended to migrate randomly after the inhibition of CXCL12/CXCR4 pathway. (E) The migration assay was performed using a specific EphB4 inhibitor. Rose diagram map demonstrated that inhibition of a EphB/ephrinB pathway blocked self-repulsive action of NSC, leading to short net distance.


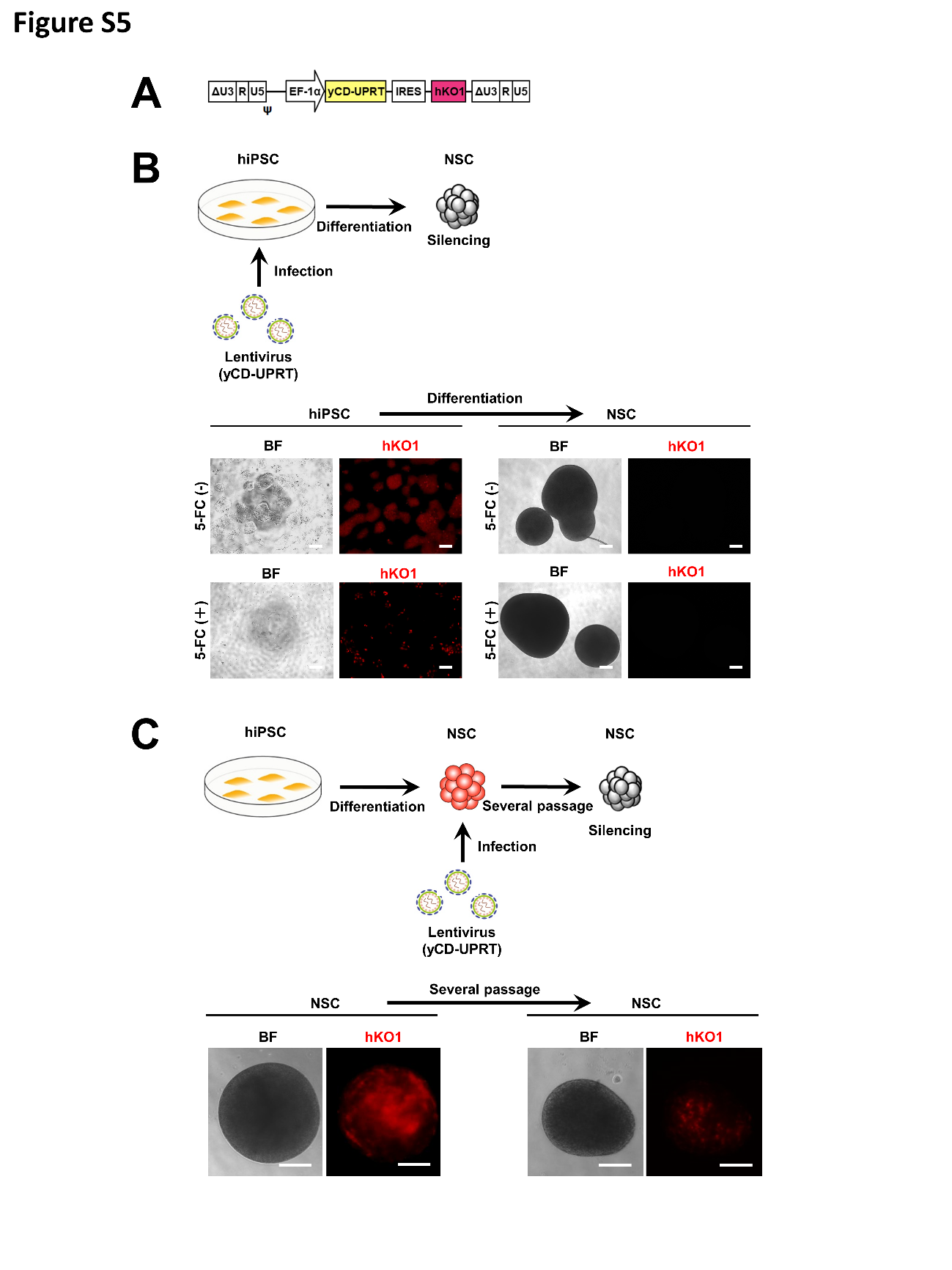


**Figure S5.** **Lentiviral vector-mediated transduction of yCD-UPRT.** (A) Schematic representation of the integrated proviral form of the lentiviral vector CSII-EF-yCD-UPRT-IRES2-hKO1. EF-1α, human elongation factor 1 α subunit promoter; IRES, internal ribosomal entry site; hKO1, humanized-codon Kusabira-Orange fluorescent protein gene; ΔU3, deletion of enhancer/promoter in the U3 region of the LTR; ψ, packaging signal. (B) hiPSCs were transduced with the lentiviral vector CSII-EF-yCD-UPRT-IRES-hKO1 at a MOI of 2. hiPSCs with yCD-UPRT-hKO1 was subsequently differentiated into NSC. Representative images of hiPSCs and NSCs with yCD-UPRT-hKO1. hiPSCs and NSCs with yCD-UPRT-hKO1 were cultured in the presence of 50 μM 5-FC for 3 days. hiPSCs were sensitive to 5-FC. However, transduced yCD-UPRT-hKO1 was silenced during neuronal differentiation of hiPSCs. hKO1 fluorescence signal was not detected in NSCs. NSCs were all 5-FC resistant. (C) NSCs derived from hiPSC were transduced with the lentiviral vector CSII-EF-yCD-UPRT-IRES-hKO1 at a MOI of 2 (CD-NSC [Lenti]). Although 70–80% of NSCs were hKO1-positive immediately after transduction, the proportion of hKO1-positive cells decreased with time, and <5% of NSCs were hKO1-positive after the second passage. Scale bar, 100 μm.


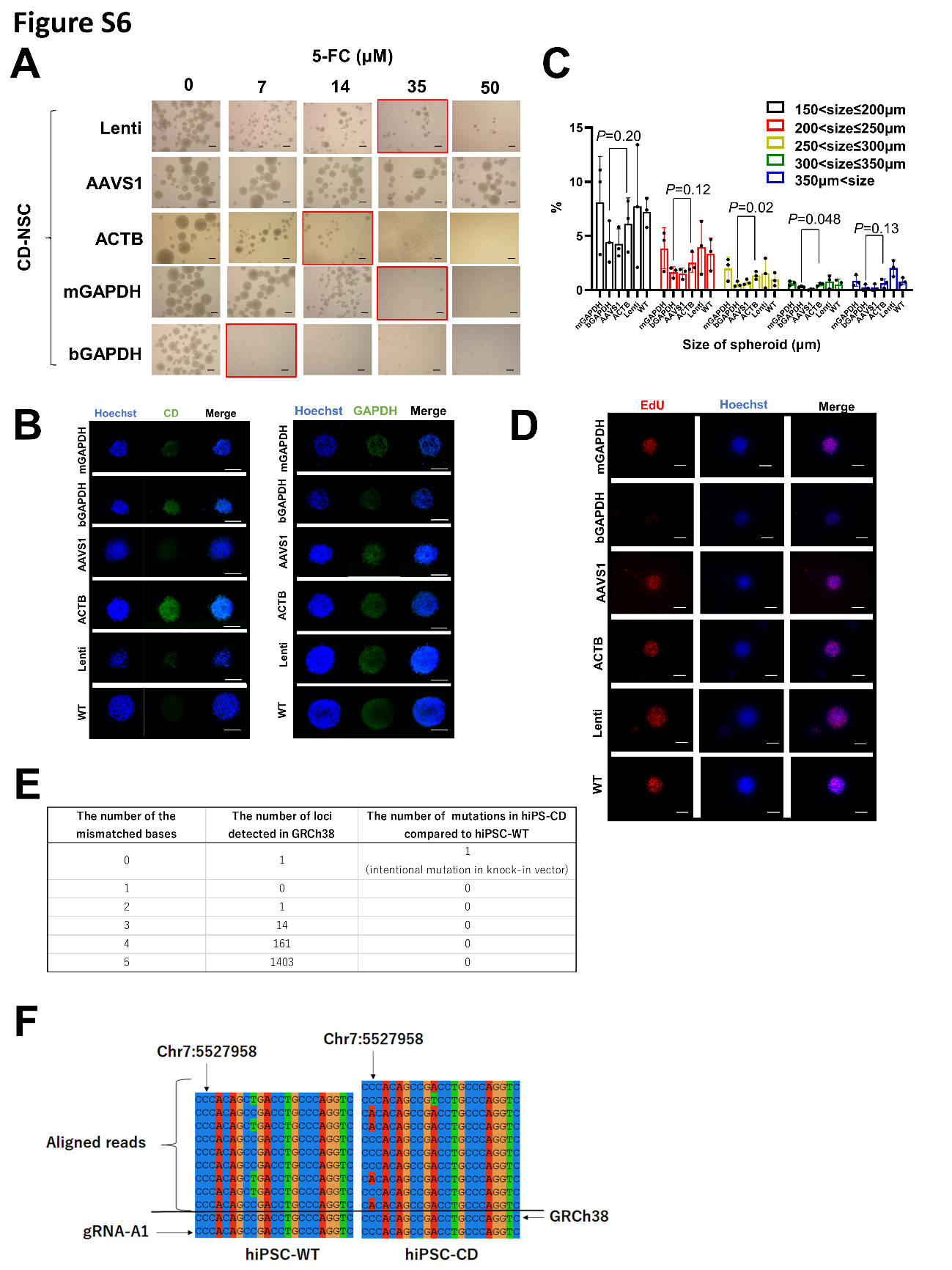


**Figure S6.** **yCD-UPRT knock-in into specific target sites.**

(A) Representative images of each CD-NSC in Figure 2B are shown. CD-NSCs were cultured in the presence of 0, 7, 14, 35 or 50 μM 5-FC for 7 days. The sensitivity to 5-FC was evaluated by CCK-8 assay. Red square, the concentration of 5-FC that achieved complete cell death. (B) Immunocytochemical analysis of neurospheres stained with anti-GAPDH antibody or anti-CD antibody and DAPI. Densitometry of the signals was evaluated. Scale bars, 100 mm. (C) Distribution of the different sizes of neurospheres and the total numbers of each CD-NSC (n = 3 independent cultures). The frequency of neurospheres larger than 150 μm is shown as the percentage of total neurospheres. WT, wild type hiPSC-derived NSCs. (D) Representative confocal images of EdU^+^ cells (red) in neurospheres. Nuclei were stained with Hoechst33258 (blue). Scale bars, 100 mm. (E) Off-target analysis of hiPSC-CD by whole-genome sequencing (WGS). (F) The aligned reads of WGS in the On-target site. The mutation of Chr7:5527958 is induced intentionally in the knock-in vector.


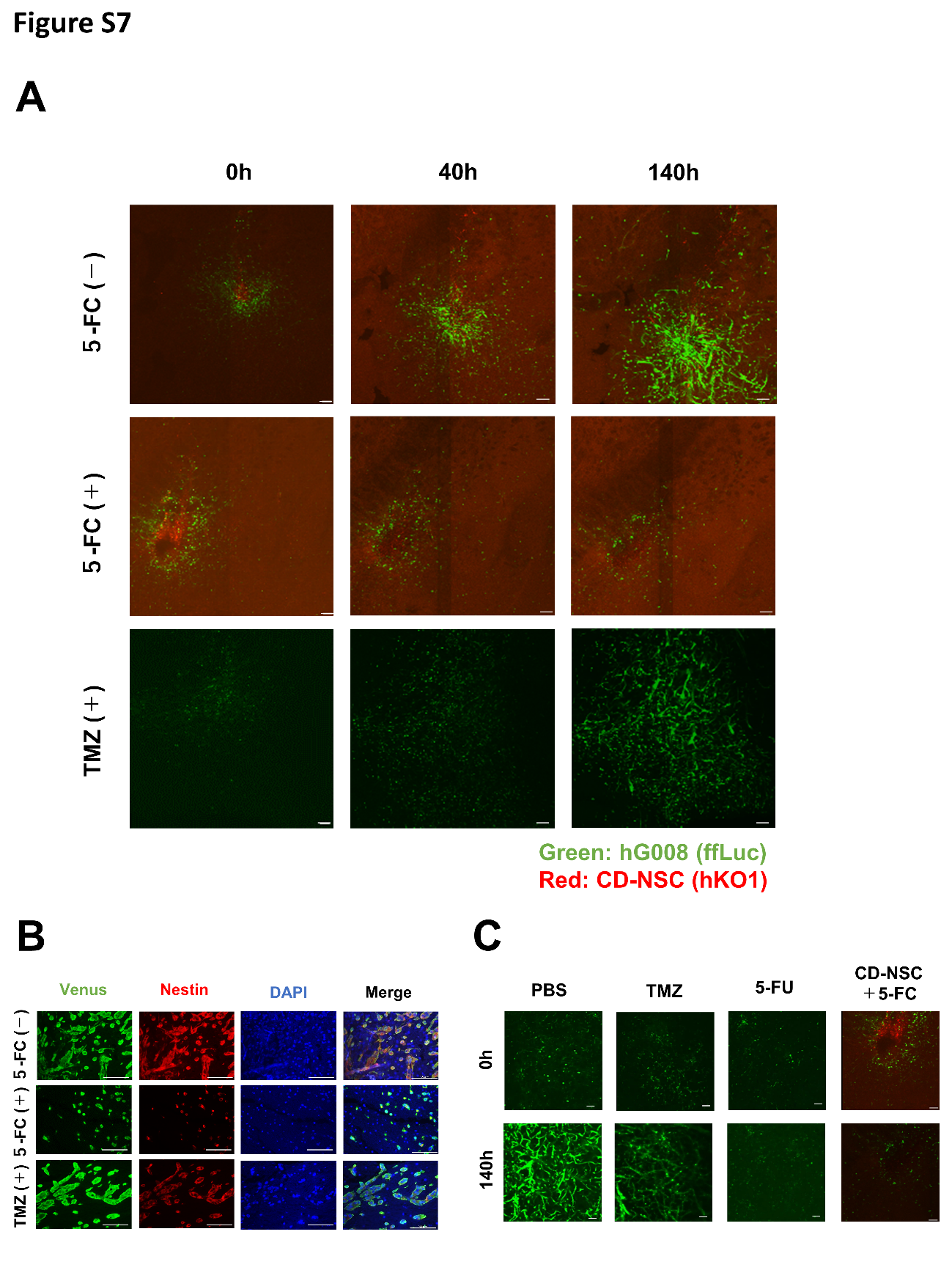


**Figure S7.** **Evaluation of anti-tumor effects using an organotypic brain slice culture.**

(A) Snapshot fluorescence images of slice cultures from brains transplanted with hG008 cells (ffLuc) and CD-NSCs (hKO1^+^) or transplanted with hG008 cells (ffLuc) alone at the indicated timepoints. In the absence of 5-FC or presence of TMZ, hG008 cells (ffLuc) showed high proliferation during the 140-hour culture period. Conversely, when treated with 5-FC, the growth of hG008 cells (ffLuc) in the slice cultures with CD-NSCs (hKO1^+^) was markedly inhibited. hG008 cells (ffLuc) began to die 48 hours after 5-FC administration. hG008 cells (ffLuc), that invaded far from the implanted site were killed by converted 5-FU released from CD-NSCs (hKO1^+^). Scale bar, 100 μm. See Additional file 9: Movie S3. (C) Slice cultures from the indicated brains after 140 hours of culture were stained with anti-GFP and anti-nestin antibodies. Scale bar,100 μm. (D) Snapshot fluorescence images of slice cultures from brains transplanted with hG008 cells (ffLuc) and CD-NSCs (hKO1^+^) treated using 5-FC or transplanted with hG008 cells (ffLuc) alone at the indicated timepoints. Snapshot fluorescence images from brains transplanted with hG008 cells (ffLuc) after TMZ or 5-FU administration. Scale bar, 100 μm.


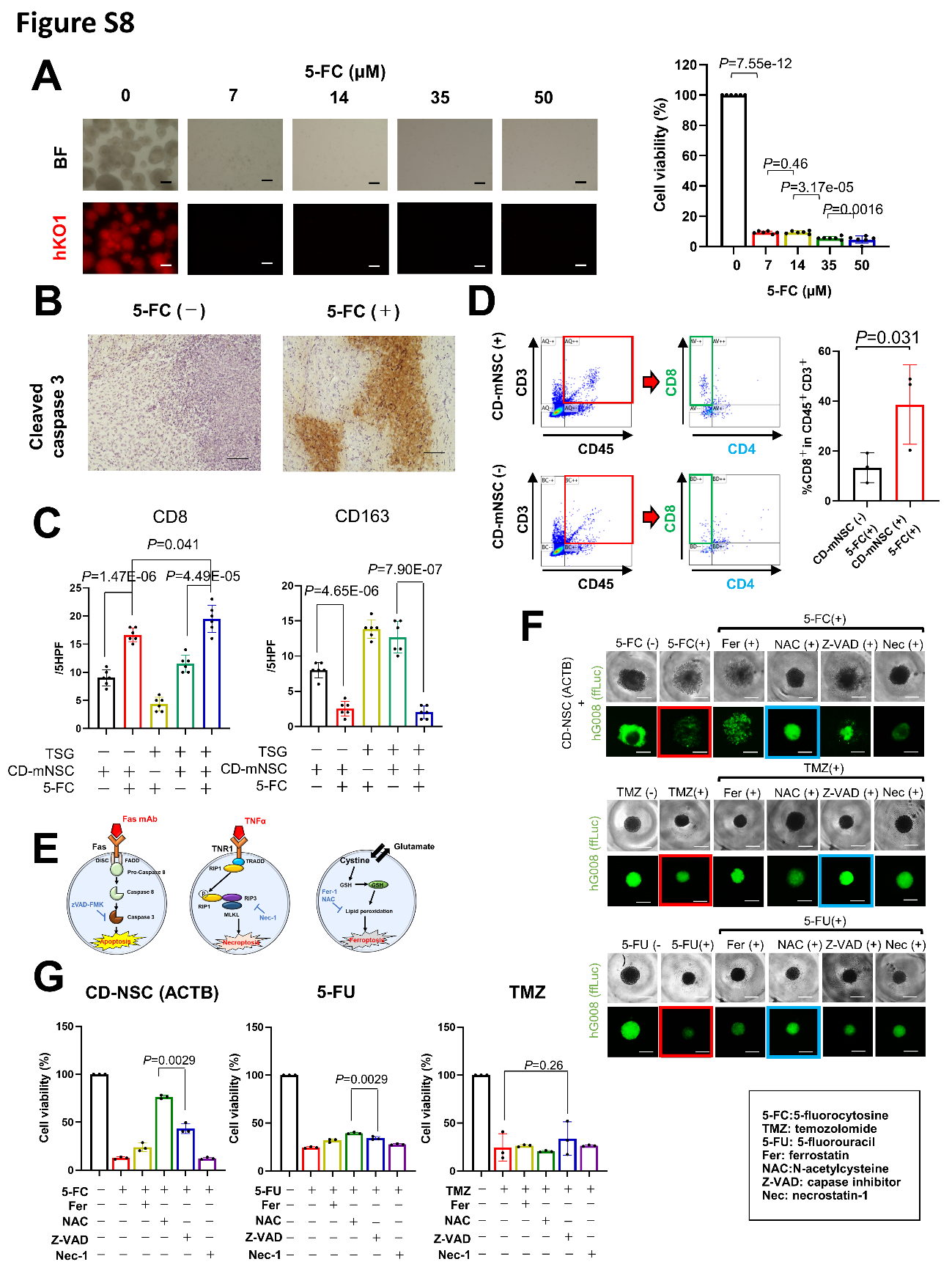


**Figure S8.** **Anti-tumor immune response and ferroptosis mediated by CD-NSCs.**

(A) Representative images of CD-mNSCs with hKO1 fluorescence. CD-mNSCs were cultured in the presence of 0, 7, 14, 35, or 50 μM 5-FC for 7 days. The sensitivity of CD-mNSCs to 5-FC was evaluated by CCK-8 assay. Scale bar, 100 μm. (B,C) Mice were euthanized at 3 weeks after the transplantation of TSG cells (ffLuc) with PBS or 5-FC administration and brain tissues were analysed. Immunohistochemical staining of Cleaved caspase-3 for the detection of apoptotic cells. Scale bar, 100 μm (B). Immunohistochemical analysis of CD8 and CD163 expressions. Only CD-mNSCs without tumor cells were implanted into the mouse brain to evaluate the direct immune responses to the iPSC-derived NSCs. For the assessment, the stained sections were screened at low-power fields (x4) and five hot spots were selected. Then, the number of positive cells was counted in high-power fields (x 40, 0.47 mm^2^; C). (D) Identification and quantification of T cells subpopulations in tumor tissues flow cytometry analysis using monoclonal antibodies against CD45, CD3, CD4, and CD8. Mice transplanted with TSG cells (ffLuc) followed by the administration of 5-FC or with TSG cells (ffLuc) and CD-mNSCs followed by the administration of 5-FC were evaluated. (E) Mechanisms of apoptosis, ferroptosis, and necroptosis. Ferroptosis is a form of regulated cell death, distinct from apoptosis, necroptosis, and autophagy. It is an iron-dependent process that results from the loss of glutathione peroxidase 4 activity and leads to the accumulation of lipid peroxidation products and reactive oxygen species. Z-VAD(OMe)-FMK (zVAD-FMK) and necrostatin-1 (Nec-1) are well-defined apoptosis and necroptosis modulators, respectively. Ferrostatin-1 (Fer-1) and N-acetylcysteine (NAC) inhibit ferroptosis. (F) Analysis of apoptosis, necroptosis, and ferroptosis induced by CD-NSC (ACTB), 5-FU, and TMZ. Representative images of hG008 cells (ffLuc) with Venus fluorescence. Fer, NAC, Z-VAD, or Nec were used. Scale bar, 100 μm. Red square, tumor cell death by the indicated drug. Blue square, rescue of tumor cell death by the indicated drug. (G) Death of hG008 (ffLuc) cells by the indicated drug was evaluated by CCK-8 assay. Data represent the mean ± SD (n =3).


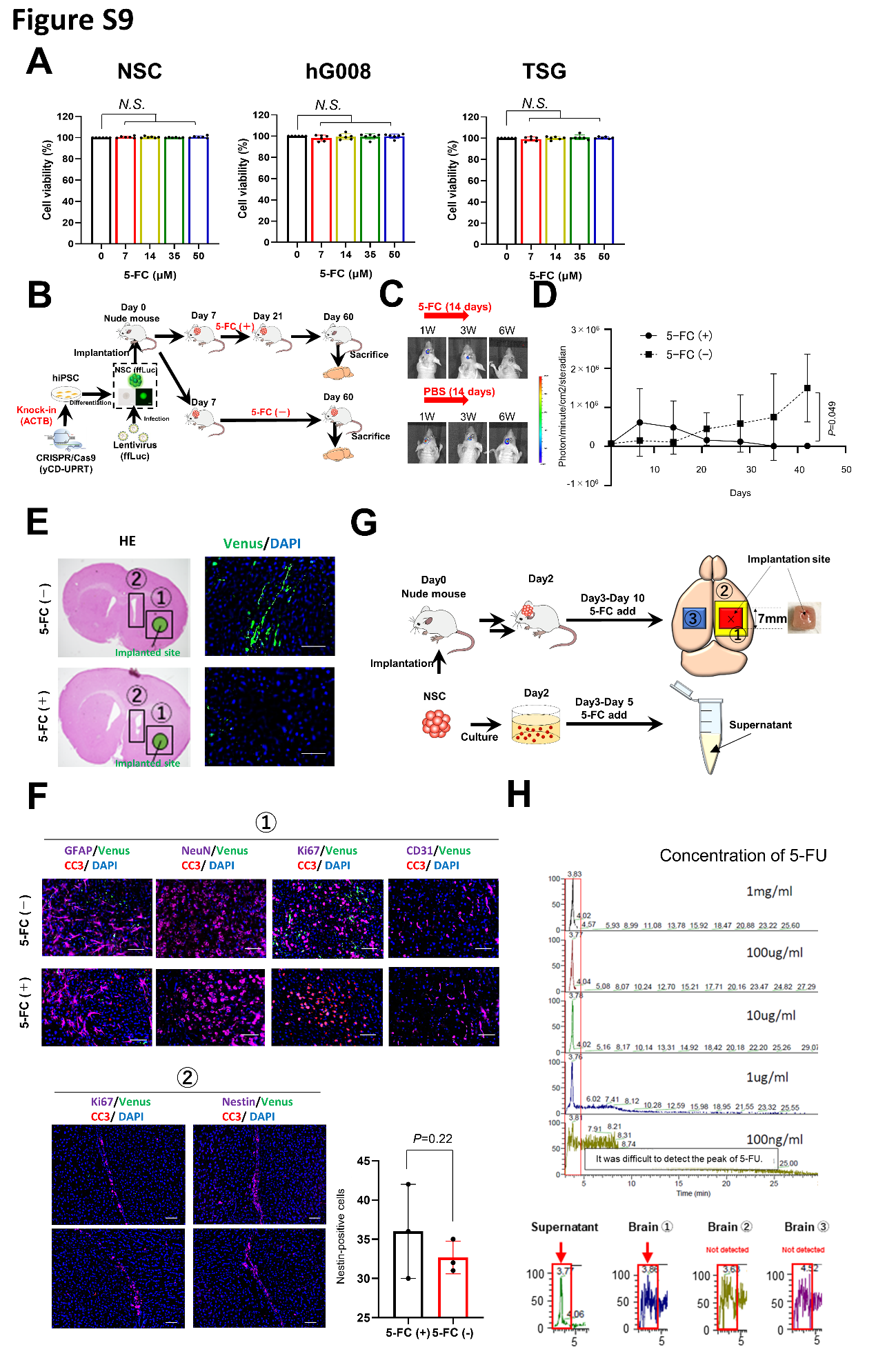


**Figure S9.** **Safety of the implanted CD-NSCs (ACTB) in normal brain parenchyma.**

(A) iPSC-NSC, hG008, or TSG was cultured in the presence of 0, 7, 14, 35 or 50 μM 5-FC for 7 days. The sensitivity to 5-FC was evaluated by CCK-8 assay. Data represent the mean ±SD (n =6).

(B) Schematic overview of the safety evaluation. CD-NSCs (ACTB) were transduced with the lentiviral vector CSII-EF-*ffLuc*. 5-FC was administered 7 days after the implantation of CD-NSCs (ACTB; ffLuc). Histological analysis was performed on Day 60 after the implantation. Mice without 5-FC administration were used as controls. (C,D) Representative BLI images and radiance intensities of mice transplanted with CD-NSCs (ACTB; ffLuc) followed by the administration of 5-FC. The mice received 5-FC once daily from Day 7 to 21. Complete disappearance of the BLI signal intensity in all mice (n=3) that received 5-FC (C). The coloured scale bar represents BLI radiance intensity in photons/minute/cm^2^/steradian. Mean signal intensities in each group after the transplantation were plotted. SD (error bars) is shown (D). (E,F) Immunohistochemical analysis of Venus expression representing CD-NSCs (ACTB; ffLuc). Brain tissues from the mice administered 5-FC or PBS at 60 days after implantation were analysed (n=3/group). Implanted area (①) and periventricular area (②) were evaluated. No CD-NSCs (ACTB; ffLuc) were observed in the implanted area (①) after 5-FC administration (E). Immunohistochemical analysis of Cleaved caspase 3, GFAP, NeuN, Ki-67, and CD31 to evaluate the influence of implanted CD-NSCs (ACTB; ffLuc) on normal brain. Proliferating mouse endogenous neural progenitor cells in the walls of lateral ventricles (②) were detected by nestin and Ki-67 staining. Nestin-positive neural progenitor cells were counted in the brain tissue with and without 5-FC administration. Scale bar, 100 μm (F). (G) Schematic overview of LC-MS. The concentration of 5-FU produced from CD-NSCs (ACTB) was measured in the supernatant and brain tissues by LC/MS. Brain tissues were obtained from the implanted area (①), ipsilateral side around the implanted area (②), contralateral side of the implanted area (③). Administration of exogenous 5-FU was used as a control. (H) LC/ MS was developed and validated to quantify 5-FU. Concentration of 5-FU (100ng/ml-1mg/ml) were evaluated. LC-MS revealed that 15 μM of 5-FU was secreted into the supernatant by CD-NSCs (ACTB). After CD-NSCs (ACTB) were implanted into normal brains, 2 μM of 5-FU was detected in a 7×7×7 mm implanted brain region (①). The retention time and peak shape (red arrow) were not detected in other areas (② and ③), which suggests a high local concentration of 5-FU.


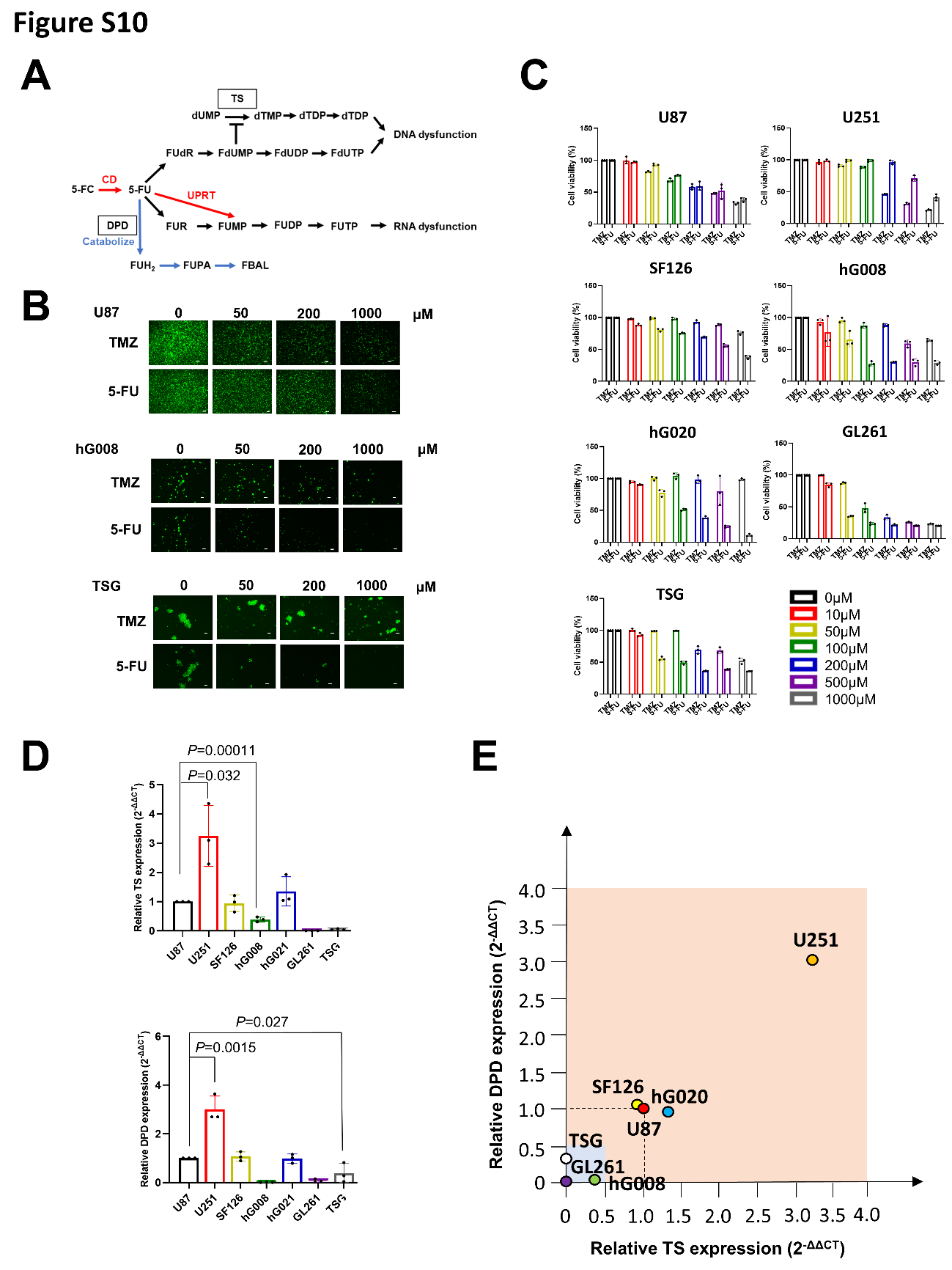


**Figure S10.** **Biomarkers to predict therapeutic response.**

(A) The metabolism of 5-FU. 5-FU is catabolised rapidly to the inactive metabolite dihydrofluorouracil (FUH2) by the DPD. The main action of 5-FU is caused by its active metabolite: 5-fluoro-uridine-5′-triphosphate (FUTP) or 5-fluoro-2′-deoxyuridine-5′-monophosphate (FdUMP). Metabolites such as FUTP can be incorporated into RNA, while FdUMP suppresses TS, an essential DNA de novo synthetic enzyme that catalyses the methylation of deoxyuridine monophosphate (dUMP) to deoxythymidine monophosphate (dTMP). (B,C) Glioma cells were cultured in the presence of 0-1000 μM 5-FU or TMZ for 7 days. Representative images of U87, hG008 and TSG are shown (B). The sensitivity to 5-FU and TMZ was evaluated by CCK-8 assay (C). Data represent the mean ±SD (n =3). (D) qRT-PCR analysis of each glioma cell for *TS* or *DPD* expression. Gene expression was normalized relative to GAPDH expression. Data represent the mean ±SD (n =3). (E) DPD expression plotted against TS expression in U87, U251, SF126, hG008, hG020, GL261, and TSG.

**3. Supplementary Tables**

**Table S1.** **Therapeutic neural stem cells used in this study.**

| **Name** | **Transduction method** | **Establishment of therapeutic NSC** |
| --- | --- | --- |
| CD-NSC (Lenti) | Lentiviral vector | NSCs derived from hiPSCs were transduced with a lentiviral vector encoding yCD-UPRT |
| CD-NSC (mGAPDH or bGAPDH*) | CRISPR/Cas9 | yCD-UPRT was inserted into monoallelic *GAPDH (mGAPDH)* or biallelic *GAPDH (bGAPDH)* gene loci using CRISPR/Cas9, and transduced hiPSCs were differentiated into NSCs |
| CD-NSC (AAVS or ACTB) | CRISPR/Cas9 | yCD-UPRT was inserted into *AAVS-1* or *ACTB* gene loci using CRISPR/Cas9, and transduced hiPSCs were differentiated into NSCs |
| CD-mNSC | Lentiviral vector | NSCs derived from miPSCs were transduced with a lentiviral vector encoding yCD-UPRT. |

bGAPDH, biallelic GAPDH; iPSCs, induced pluripotent stem cells; m, mouse; mGAPDH, monoallelic GAPDH; NCS, neural stem cell; yCD, yeast cytosine deaminase; UPRT, uracil phosphoribosyltransferase.

*CD-NSCs (bGAPDH) were established to increase yCD-UPRT gene expression, because CD-NSCs (mGAPDH) did not show marked sensitivity to 5-FC.

**Table S2.** **Sequences of CRISPR gRNAs used in this study.**

| Name | gRNA sequence (5'-3') 　PAM | Locus |
| --- | --- | --- |
| gRNA-G1* | GTGCAGGGTCTGGCGCCCTC　TGG | chr12 + 6538031-6538054 |
| gRNA-A1* | GACCTGGGCAGGTCGGCTGT　GGG | chr7 – 5527956-5527979 |
| gRNA-T2* | GGGGCCACTAGGGACAGGAT　TGG | chr19 – 55115748-55115771 |

gRNA, guide RNA; PAM, protospacer adjacent motif.

*gRNA-G1: guide RNA for GAPDH locus; gRNA-A1: guide RNA for ACTB locus; gRNA-T2: guide RNA for AAVS1 locus.

**Table S3.** **Primer sequences used to quantify mRNA.**

| Gene | Forward (5'-3') | Reverse (5'-3') |
| --- | --- | --- |
| GAPDH | GTCCACTGGCGTCTTCACCA | GTGGCAGTGATGGCATGGAC |
| β-actin | GATCAAGATCATTGCTCCTCCT | GGGTGTAACGCAACTAAGTCA |
| CD  TS  DPD | CACCATGGTCACAGGAGGCAT  GAATCACATCGAGCCACTGAAA  AGGACGCAAGGAGGGTTTG | TTAGACACAGTAGTATCTGTC  CAGCCCAACCCCTAAAGACTGA  GTCCGCCGAGTCCTTACTGA |

CD, cytosine deaminase; DPD, dihydroprimidine dehydrogenase; TS, thymidylate synthase

**Table S4. All relevant statistical comparisons**

See additional excel file.

**4. Supplementary Movies**

**Movie S1:** **Tumor-trophic migratory capacity of iPSC-NSCs for diffusely infiltrative hG008 cells.**

Time-lapse imaging of slice cultures from a brain transplanted with diffusely infiltrative hG008 cells (ffLuc) together with CD-NSCs (hKO1^+^) during a 140-hour culture period. Images were captured every 20 min (related to Figure S7B).

**Movie S2:** **Tumor-supporting effect of MSCs for U87 cells.**

Time-lapse imaging of slice cultures from a brain transplanted with hG008 cells (ffLuc) together with BMSC2 (hKO1^+^) or iPSC-NSCs (hKO1^+^; related to Figure S3A).

**Movie S3:** **Anti-tumor effect of CD-NSC on hG008 cells.**

Time-lapse imaging of slice cultures from a brain transplanted with hG008 cells (ffLuc) together with CD-NSCs (hKO1^+^) treated with 5-FC or PBS (related to Figure S7B).

**5. References**

61. Stupp R, Mason WP, van den Bent MJ, et al. Radiotherapy plus concomitant and adjuvant temozolomide for glioblastoma. N Engl J Med. 2005;352: 987-996.

62. Kase Y, Otsu K, Shimazaki T, Okano H. Involvement of p38 in Age-Related Decline in Adult Neurogenesis via Modulation of Wnt Signaling. Stem Cell Reports 2019;12:1313-1328.

63. Chen LY, Wei KC, Huang AC, et al. RNASEQR--a streamlined and accurate RNA-seq sequence analysis program. Nucleic Acids Res 2012;40: e42.

64. Martin M. Cutadapt removes adapter sequences from high-throughput sequencing reads. EMBnet. Journal 2019;17:10-12.

65. Patro R, Duggal G, Love M, Irizarry R, Kingsford C. GSalmon provides fast and bias-aware quantification of transcript expression. Nat Methods 2017;14:417-419.

66. Love M, Huber W, Anders S. Moderated estimation of fold change and dispersion for RNA-seq data with DESeq2. Genome Biol 2014;15:550.

67. Yu G, Wang LG, Han Y. He QY. clusterProfiler: an R package for comparing biological themes among gene clusters. OMICS 2012;16:284-287.

68. Camp JG, Sekine K, Gerber T, et al. Multilineage communication regulates human liver bud development from pluripotency. Nature 2017;546: 533-538.

69. Ramilowski JA, Goldberg T, Harshbarger J, et al. A draft network of ligand-receptor-mediated multicellular signaling in human. Nat Commun 2015;22:7866.

70. Kania A, Klein R. Mechanisms of ephrin-Eph signalling in development, physiology and disease. Nat Rev Mol Cell Biol. 2016;17:240-256.

71. Chung K, Wallace J, Kim SY, et al. Structural and molecular interrogation of intact biological systems. Nature 2013;497:332-337.
